# A Review of CO_2_ Electroreduction to Ethanol: C–C Coupling Mechanistic Insights and Catalyst Design

**DOI:** 10.1007/s40820-026-02159-y

**Published:** 2026-04-03

**Authors:** Fang Zhao, Bo Huang, Yingzheng Zhang, Tianxin Wei, Jiatao Zhang, Di Zhao

**Affiliations:** Key Laboratory of Cluster Science, Beijing Key Laboratory of Construction-Tailorable Advanced Functional Materials and Green Applications, School of Chemistry and Chemical Engineering, Beijing Institute of Technology, Beijing, 100081 People’s Republic of China

**Keywords:** CO_2_ electroreduction, Ethanol, Dynamic mechanism, Electrocatalysts, Optimization strategy

## Abstract

As a major contributor to climate change, CO_2_ has imposed severe detrimental effects on global ecosystems. Among various CO_2_ conversion strategies, the electrocatalytic CO_2_ reduction reaction (eCO_2_RR) stands out due to its ability to operate under mild conditions using renewable electricity. Compared to gaseous C_2_ products such as ethylene, ethanol as a liquid fuel demonstrates greater economic potential and broader market prospects. In recent years, copper-based electrocatalysts have emerged as leading materials for the electrochemical conversion of CO_2_-to-ethanol. Meanwhile, a number of non-copper-based electrocatalysts have also been developed to produce ethanol via C–C coupling pathways distinct from those on Cu-based materials. However, few reviews have systematically addressed the reaction mechanisms and material design principles specific to ethanol production through eCO_2_RR. In this review, we highlight the most recent advancements in this field of study. We begin by assessing the economic viability of ethanol as a CO_2_ reduction product. This is followed by a systematic summary of the reaction mechanisms and advanced characterization methods involved in ethanol production via eCO_2_RR across various pathways. Next, we discuss and compare the catalytic active sites and key electrochemical performance metrics for ethanol generation on different types of electrocatalysts. Finally, we propose several promising strategies to guide the rational design and synthesis of next-generation high-performance electrocatalysts for selective ethanol production. This review comprehensively summarizes the latest research progress in the field of ethanol production via eCO_2_RR from multiple dimensions, including the economic value of ethanol, reaction mechanisms, an introduction to various electrocatalysts and strategies for improving electrocatalysts. It not only promotes in-depth basic research, but also provides theoretical guidance for electrocatalyst design, reaction condition optimization, and industrial applications, making it of great research value and practical significance.

## Highlights

- Focusing on the economic viability of ethanol products and the application value of eCO_2_RR, this research anchors its core from an industrial conversion perspective, combining academic depth with practical orientation.
- Systematically integrating multipath reaction mechanisms, advanced characterization techniques, and the active sites and performance metrics of various electrocatalysts, a comprehensive knowledge framework has been established.
- Proposing a rational design strategy for next-generation highly selective ethanol synthesis electrocatalysts, this work provides a clear research direction for this field.

## Graphical abstract

**Figure:**
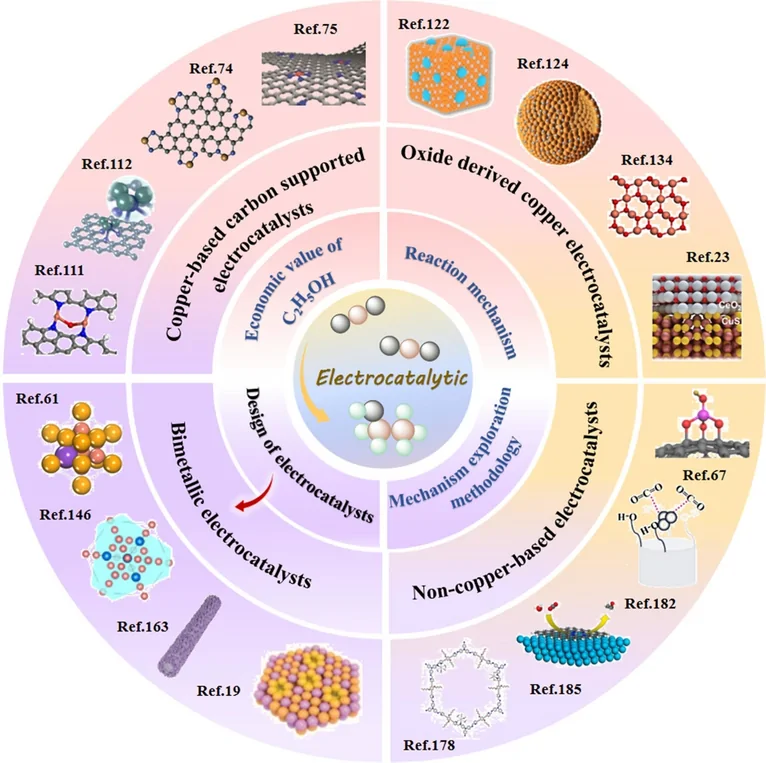

## Introduction

Since the industrial revolution, fossil fuels have dominated global energy systems, progressively displacing traditional energy sources [1–5]. However, combustion-derived CO_2_ emissions have triggered severe ecological crises—including global warming, sea-level rise, and disruption of natural carbon cycles—compromising ecosystem stability and human health [6–10]. These challenges underscore the critical need for advanced CO_2_ management strategies. Among emerging CO_2_ conversion technologies (e.g., photocatalysis, hydrogenation, biocatalysis, and thermal conversion), electrocatalytic CO_2_ reduction (eCO_2_RR) stands out by utilizing renewable electricity to transform CO_2_ into value-added products [11–15]. This process involves multiproton/electron transfers, generating diverse output products including hydrocarbons, formate, carbon monoxide, and alcohols, with selectivity governed by electrocatalyst design and operational parameters [16–18].

Ethanol, a principal C_2_ product of eCO_2_RR, is not only widely used, but also can meet the long-term, large-scale energy storage and convenient transportation, and thus has attracted wide attention in the fields of energy conversion and environmental protection [19–22]. First, as a liquid fuel, ethanol has a high-energy density and is easy to store and transport, which can reduce dependence on fossil fuels. Secondly, the production process of ethanol can realize carbon recycling through eCO_2_RR, which can effectively reduce greenhouse gas emissions and help achieve the goal of carbon neutrality. Moreover, ethanol serves as a versatile platform molecule with extensive applications across chemical synthesis, pharmaceutical manufacturing, and renewable fuel additives. The expanding market demand further accelerates research efforts toward efficient ethanol production via eCO_2_RR [23–25]. In terms of the reaction mechanism, the generation of ethanol involves the transfer of 12 electrons and the formation of C–C bonds, a process that is not only scientifically challenging, but also provides a broad research space for the design and optimization of electrocatalysts. Therefore, ethanol is significantly higher than C_1_ products in both industrial and scientific research value, making it an ideal product that most researchers want to pursue [19, 26].

**Fig. 1:**
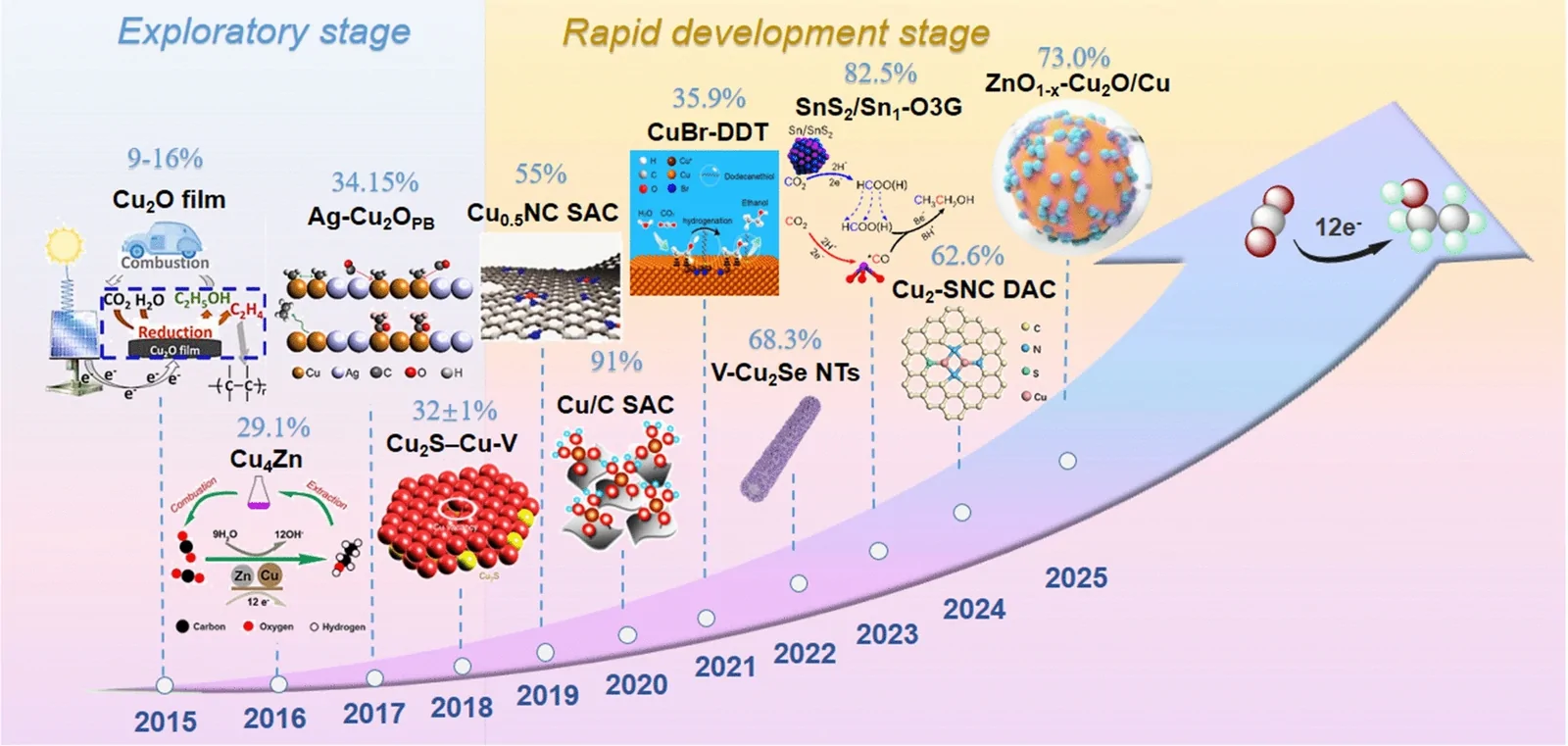
Ethanol production timeline via eCO_2_RR

As shown in Fig. 1, at present, the eCO_2_RR system for the selective generation of ethanol has been extensively researched, and the design of electrocatalysts has also advanced significantly [23, 27–33]. In terms of the reaction mechanism, the researchers revealed the key intermediates and reaction pathways for the productions of ethanol from eCO_2_RR by in situ spectroscopic techniques and density functional theory (DFT) calculations. It is generally believed that CO_2_ is first reduced to *CO intermediates or *CH_2_ intermediates, followed by the formation of key intermediates such as *COCOH or *CH_2_CHO via C–C coupling, and finally hydrogenated to produce ethanol. In terms of electrocatalyst design, Cu-based electrocatalysts are considered to be the most promising materials for eCO_2_RR ethanol production due to their unique electronic structure and catalytic properties. Several studies have demonstrated that the crystalline surface, morphology, oxidation state, and surface defects of copper have a significant effect on the selectivity of ethanol. For example, by modulating the crystalline surface exposure or building nanostructures of copper electrocatalysts, the C–C coupling reaction can be effectively promoted, thus improving the ethanol generation efficiency. In addition, Cu-based bimetallic electrocatalysts (e.g., CuAg, Cu–Zn, etc.) have also been widely studied to further enhance the ethanol selectivity through synergistic effects. More importantly, in recent years, studies have been successively reported on ethanol production over non-Cu-based electrocatalysts through a unique mechanism, which represents a significant advancement in the field of eCO_2_RR-to-ethanol. Despite important progress in eCO_2_RR ethanol production research, a number of challenges remain. On the one hand, the synthesis of ethanol through eCO_2_RR process still suffers from high overpotential, low Faradaic efficiency (FE), and poor yield rate [34, 35]. On the other hand, the hydrogen evolution reaction’s (HER) equilibrium potential is comparable to that of eCO_2_RR, resulting in HER being an unavoidably competitive reaction [36]. Because the multielectron process is very sensitive to the inherent characteristics of the electrocatalytic materials and electrolyte components, it is necessary to design an efficient catalytic system of eCO_2_RR with high selectivity, high Faradaic efficiency, and low overpotential for ethanol.

**Fig. 2:**
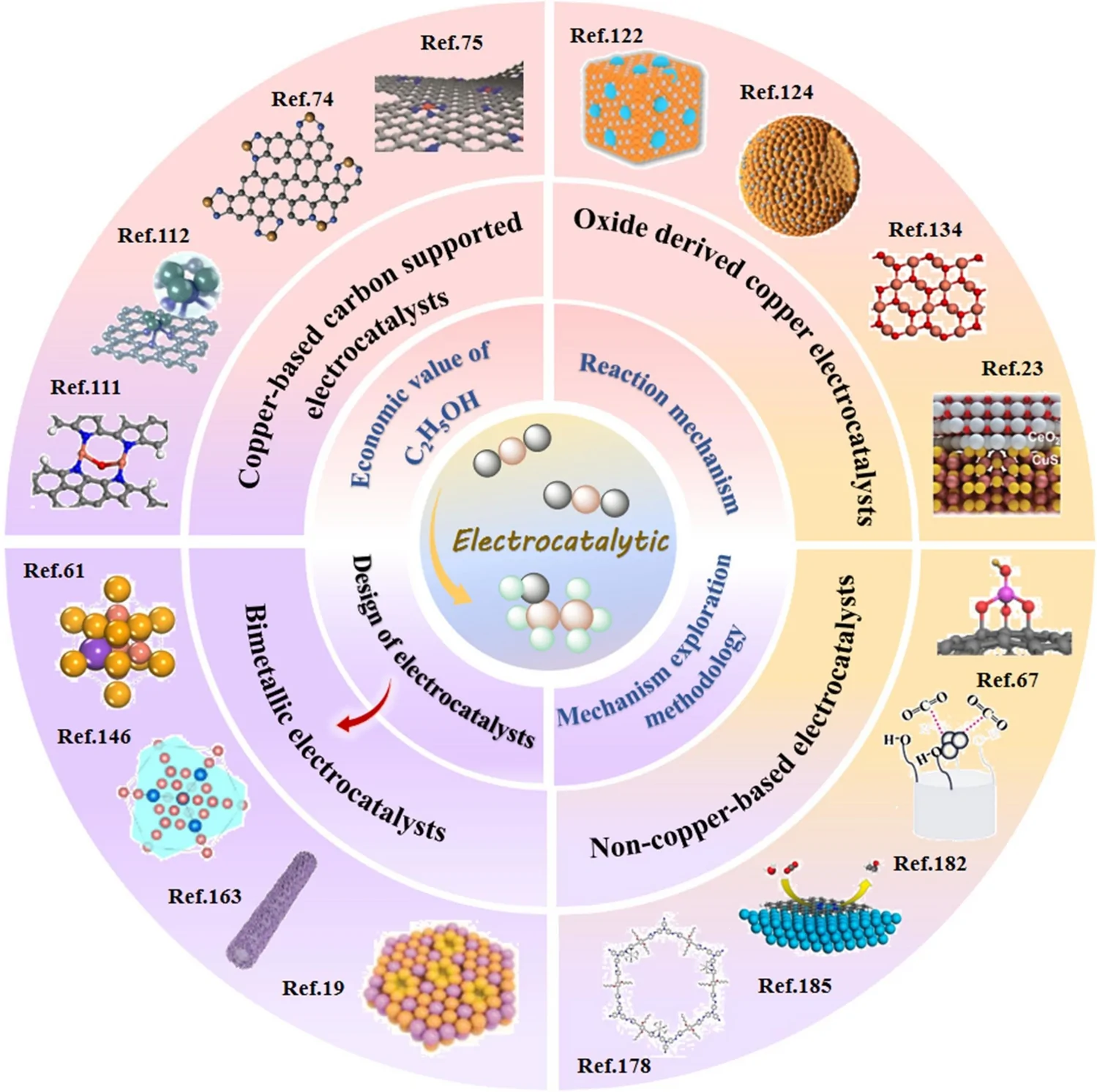
Overview of themes discussed in this review

This review provides a comprehensive overview of recent advances in electrocatalytic CO_2_ reduction for selective ethanol production (Fig. 2). We first analyze the economic viability and environmental necessity of CO_2_-to-ethanol conversion, followed by systematic examination of: (1) reaction mechanisms and pathways for ethanol formation; (2) structure–activity relationships of catalytic sites on copper-based and non-copper-based electrocatalysts; (3) electrochemical performance metrics (Faradaic efficiency, potential, current density) across material systems. Building on these analyses, we propose targeted design strategies for next-generation electrocatalysts, including atomic-scale engineering, tandem catalysis, and device optimization. This work not only identifies key scientific challenges and technical bottlenecks in the field, but also establishes foundational principles for electrocatalyst design, reaction condition optimization, and industrial implementation, offering both theoretical guidance and practical pathways toward sustainable ethanol production.

## Economic Analysis for CO_2_-to-Ethanol

Against the backdrop of continued growth in global energy demand, the environmental pressures and energy crisis brought by using of traditional fossil fuels have become particularly pronounced. Ethanol fuels have received unprecedented attention due to their sustainable and environmentally friendly nature, as well as the high value of ethanol itself [37, 38]. As most of the current supply of ethanol comes from biomass, the use of energy-efficient, low-cost, carbon–neutral electrochemical processes to convert CO_2_ directly into ethanol can help reduce ethanol and food costs, as well as reduce the impact of CO_2_ on the global ecosystem [39–41].

**Fig. 3:**
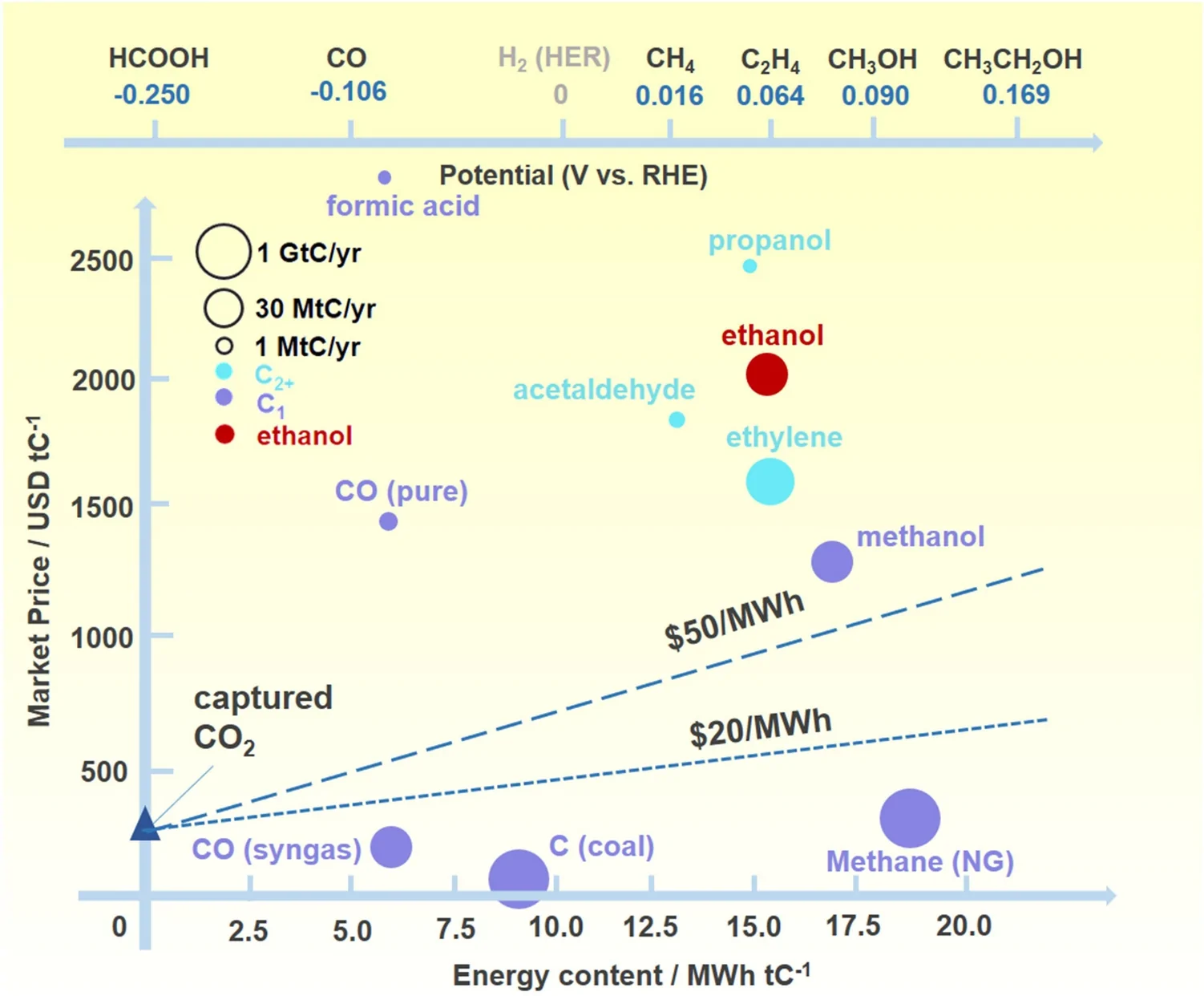
Thermodynamic and economic landscape of CO_2_ electrolysis products. Standard reduction potentials for the formation of various products from CO_2_, highlighting the multielectron transfer required for ethanol (upper panel). Market price of selected CO_2_ reduction products plotted against the electrical energy required for their production (normalized per kg). The lines represent the minimum cost considering only energy and CO_2_ feedstock [45]. This analysis underscores that ethanol represents a particularly economically viable target among multicarbon products

From the perspective of techno-economic analysis, Fig. 3 demonstrates that ethanol, as a C_2_ product, holds a higher market value in the new energy and chemical industries due to its higher volumetric energy density and larger market size compared to C_1_ products [42]. Secondly, factors such as the market supply and demand for ethanol are also a major reflection of the economic value of eCO_2_RR. For example, at present, ethanol is a commodity with great market demand, and in the first half of 2024, China’s fuel ethanol production was 3.72 million tons, a year-on-year increase of 22%. As for the market demand, the demand for ethanol in the food and beverage sector has declined more significantly, while the downstream demand in the chemical sector has increased; thus, ethanol occupies an irreplaceable position in the energy and chemical sector [43]. In addition, under relatively standard hydrogen electrode conditions, compared to the 2-electron transfer reaction, CO_2_ electroreduction to ethanol is a reaction involving a 12-electron transfer process, which requires more electrical energy consumption, and with the change in the number of electrons transferred, the resulting product also changes, and the corresponding market price is calculated after normalization according to the amount of electrical energy required for each reduction product [44]. Assuming an industrial electricity cost of $0.05 kW^−1^ h^−1^, the minimum cost of ethanol production from eCO_2_RR is approximately $0.40 kg^−1^. It is important to note that the above calculations are based on a representation of the ethanol yield that can be generated per kWh of electricity used. Therefore, given the market price of the product and the cost of electricity, ethanol is the most promising multicarbon product. Furthermore, the current density of electrocatalysis is another key metric for evaluating the production of commercially valuable products from eCO_2_RR, with the maximum sub-current density of ethylene at η ≈ 640 mV ranging from 1100 to 1350 mA cm^−2^. The sub-current density of ethanol (~ 160 mA cm^−2^ at *η* = 770 mV) is much lower, and for commercial application of eCO_2_RR technology, partial current densities in the range of 100–1000 mA cm^−2^ are highly desirable, which reduces reactor size and capital cost. Thus, electrocatalytic CO_2_-to-ethanol conversion represents the most economically and technologically viable pathway for achieving multicarbon product synthesis via CO_2_ electroreduction.

## Understanding of eCO_2_RR-to-Ethanol

### Reaction Pathways

**Fig. 4:**
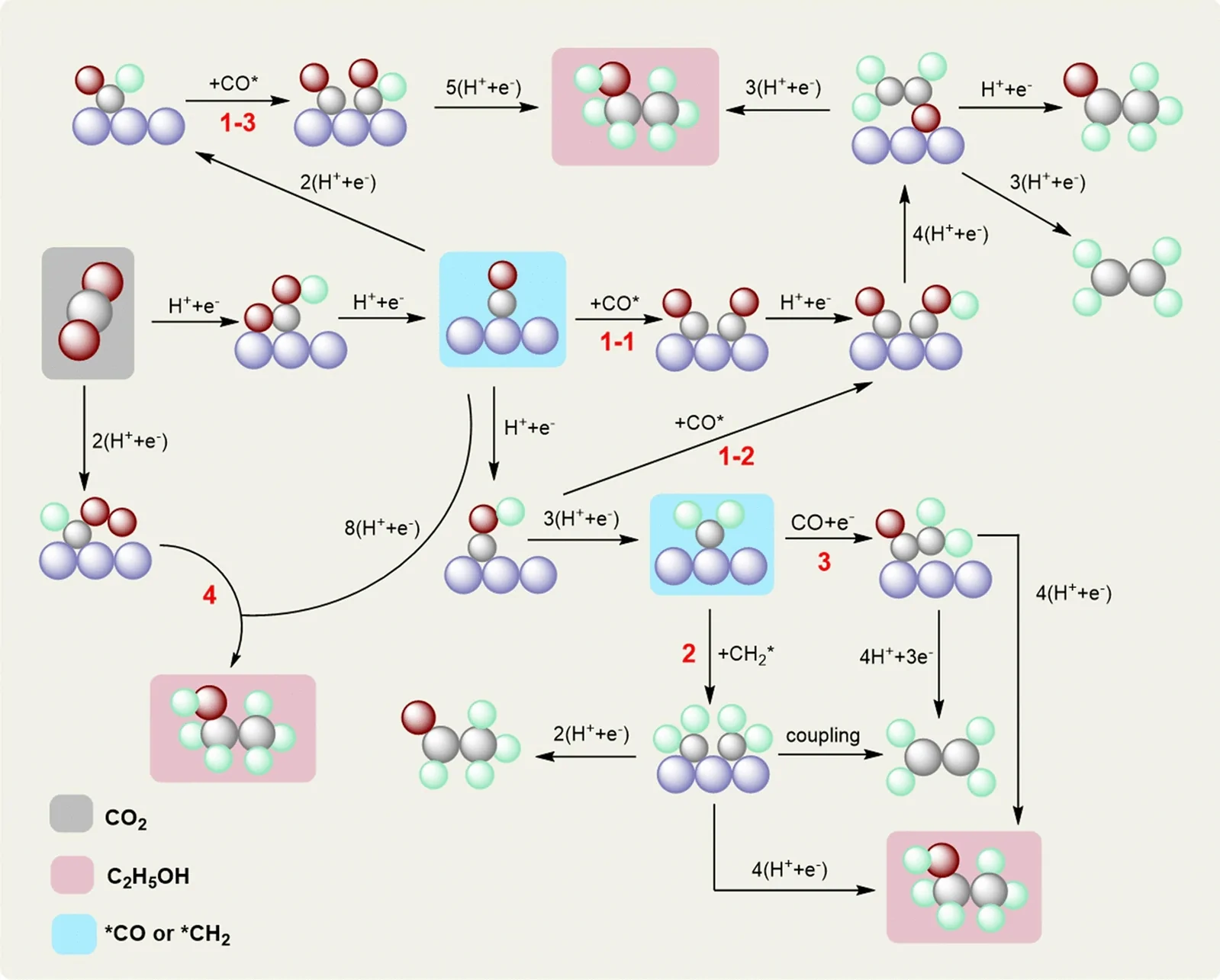
Several possible reaction pathways for the eCO_2_RR to C_2_H_5_OH

The electrochemical reduction of CO_2_ (eCO_2_RR) is a typical three-phase reaction involving gas feedstock, liquid electrolyte, and solid electrode. Due to its intricate thermodynamic and kinetic processes, ethanol is generated from different chemical reaction routes and intermediates in the process of CO_2_ electroreduction [46–48]. According to most studies reported so far, both ethanol and ethylene should be derived from a common reaction intermediate. This common reaction intermediate may be *OC-CO, or it may be one of the reaction intermediates after *OC–CO, in which different follow-up processes will determine the synthesis of ethylene and ethanol [49]. Furthermore, there is experimental evidence that acetaldehyde can also be reduced to ethanol. As shown in Fig. 4, this pathway is via *CO co-dimerization with electron transfer to form *C_2_O_2_^−^key intermediates, followed by protonation to *COCOH, and finally by reduction to generate ethylene, acetaldehyde, and ethanol products [37, 50, 51]. In contrast, the other pathway begins with four-electron reductive dehydration to generate *CH_2_, which may be followed by the coupling of two *CH_2_, which undergoes electronic reductive dehydration to generate ethylene, which also generates ethanol [52]. Or CO is inserted into *CH_2_, which can be reduced to produce acetaldehyde and *OCH_2_CH_3_ intermediates, and *OCH_2_CH_3_ intermediates are further reduced to produce ethanol [53, 54]. Subsequently, based on the different ways in which the intermediates participate in C–C coupling, the mechanisms reported for electrocatalytic CO_2_-to-ethanol are classified as follows:

#### Pathway of *CO Dimerization

Many studies have suggested that CO and HCOOH are the initial products of the electrochemical reduction of CO_2_ [55]. Whereas HCOOH usually does not undergo further electrochemical reduction, the adsorption of *CO is therefore a crucial step in the generation of ethanol. The stepwise coupling of *CO through the proton-coupled electron transfer (PCET) step enables ethanol to be obtained from different pathways, the most common being the direct coupling of two *CO intermediates [56]. In addition, there are two types of asymmetric coupling: *CO with *COH and *CO with *CHO [57, 58].

1. The pathway of *CO dimerization: This pathway initiates when two *CO molecules adsorb onto adjacent active sites on the electrocatalyst surface. Under the synergistic action of electrons, C–C bond coupling then occurs, leading to the formation of a symmetric *COCO intermediate. This coupling step is generally rate-determining. Subsequently, the *COCO intermediate accepts a proton and an electron. This results in hydrogenation at the terminal oxygen atom, yielding the hydroxyl-group-containing *COCOH intermediate [59]. Then, *COCOH undergoes sequential hydrogenation to gradually construct the ethanol carbon chain, converting through intermediates such as *CHCOH and *HCCHOH, ultimately generating *OCH_2_CH_3_ and desorbing as C_2_H_5_OH.
2. The coupling pathway of *CO and *COH: In this process, a proton first forms *COH with *CO, then dimerizes with another *CO to form *COCOH, followed by successive PCET steps and dehydrogenation to form *CHCH_2_OH, followed by another PCET step to obtain *CH_2_CH_2_OH, and then *CH_2_CH_2_OH undergoes a protonation process to obtain C_2_H_5_OH [57, 60].
3. The coupling pathway of *CO and *CHO: In this pathway, some CO_2_ molecules undergo CO_2_^−^ protonation to form HCOO^−^, which is then converted to the *CHO intermediate via H_2_COO^−^. Another portion undergoes deprotonation of the CO_2_^−^ and *COOH intermediates to generate *CO. When *CO and *CHO adsorb onto adjacent active sites, the C–C coupling process is initiated, forming the *COCHO intermediate [58, 61]. Subsequently, *COCHO first accepts a proton and an electron to form *HOCH_2_CO, which then undergoes further conversion via the *HOCH_2_CHO intermediate, followed by hydrogenation to form *HOCH_2_CH_2_O, and finally desorbs to form C_2_H_5_OH [62].

#### Pathway of Coupling of Two *CH_***2***_*** Species***

Not only can symmetric coupling be achieved via *CO, but another *CH_2_ intermediate can also be used to obtain C_2_H_5_OH via a symmetric coupling pathway. Whereas the pathway for the productions of C_2_H_5_OH is complex and each step of the protonation process involves a different pathway, only the more typical pathways are described here. Typically, it is the CO_2_ first generated *CO can be protonated to form absorbed *COH or *CHO, and then, *CHO and *CO undergo several PECT steps to form *CH_2_CHO [63, 64]. Further hydrogenation of *CH_2_CHO determines the selectivity between C_2_H_5_OH and C_2_H_4_, and if *CH_2_CHO undergoes a subsequent two-step PECT step it can form *CH_3_CH_2_O intermediate, and the final *CH_3_CH_2_O protonation process can produce either C_2_H_6_ or C_2_H_5_OH, depending on which product is favored by the energy potential of the two [65].

#### Pathway of Insertion of CO in *CH_***2***_

In addition to the above symmetric C–C coupling mechanism, the asymmetric *CH_2_^−^*CO coupling mechanism can also accomplish high selectivity for C_2_H_5_OH, specifically, a unique mechanism in which the high coverage of *CH_2_ intermediates at the working electrode and the locally higher CO concentration induces C–C coupling. In order to generate *CH_2_ and realize asymmetric coupling, it is usually necessary to introduce metal ions with different adsorption properties for the two intermediates on the active site, one of which replaces the copper ion on the conventional symmetric bimetallic site, which provides the necessary prerequisites for asymmetric coupling on the heterometallic bimetallic active site, and after that asymmetric C–C coupling occurs by electrophilic attack on the *CH_2_ intermediates by CO, CO* insertion into CH_2_* to form *CH_2_CO intermediate, and further hydrogenation of *CH_2_CO intermediate to form C_2_H_5_OH [66].

#### Pathway of Coupling of *CO(OH) and *CHO

Although the vast majority of reported C–C coupling mechanisms follow the conventional pathways described above, recent studies have revealed alternative coupling routes during ethanol production on non-copper-based electrocatalysts. These emerging mechanisms demonstrate distinct C–C bond formation pathways that deviate fundamentally from copper-catalyzed coupling processes. One of the most exemplary is a Sn-based copper-free electrocatalyst reported by Huang et al. [67]. The formation of C_2_H_5_OH on this electrocatalyst is due to the coupling of two intermediates related to CO and HCOOH, as shown in Fig. 4, where asymmetric doubly active centers consisting of Sn and oxygen atoms adsorb *CHO and *CO(OH) carbon-based intermediates, respectively, which leads to the formation of C_2_H_5_OH via a special *CHO–*CO(OH) coupling pathway to promote C_2_H_5_OH production.

### Dynamic Mechanisms of Electrocatalyst

It is well known that the Cu element is still one of the most reported and promising elements for the production of multicarbon products such as ethanol via eCO_2_RR [68, 69]. While isolated Cu sites usually cannot be used as active sites to promote the catalytic reaction, the dimensional and structural changes of Cu-based electrocatalysts are particularly crucial in the electrochemical reconfiguration process of eCO_2_RR, which can have a great impact on the selectivity of ethanol [42, 70, 71]. By studying the mechanism and kinetic process of the material surface reconstruction, the conformational relationship on the electrocatalyst surface can be revealed and provide theoretical guidance for the design and synthesis of electrocatalysts. In addition, by modulating the reaction conditions and the composition of the electrocatalysts, the control of the electrocatalyst surface reconstruction can be achieved to optimize the efficiency and selectivity of eCO_2_RR [72, 73]. This subsection provides a brief overview of the dynamics of the active sites during eCO_2_RR.

**Fig. 5:**
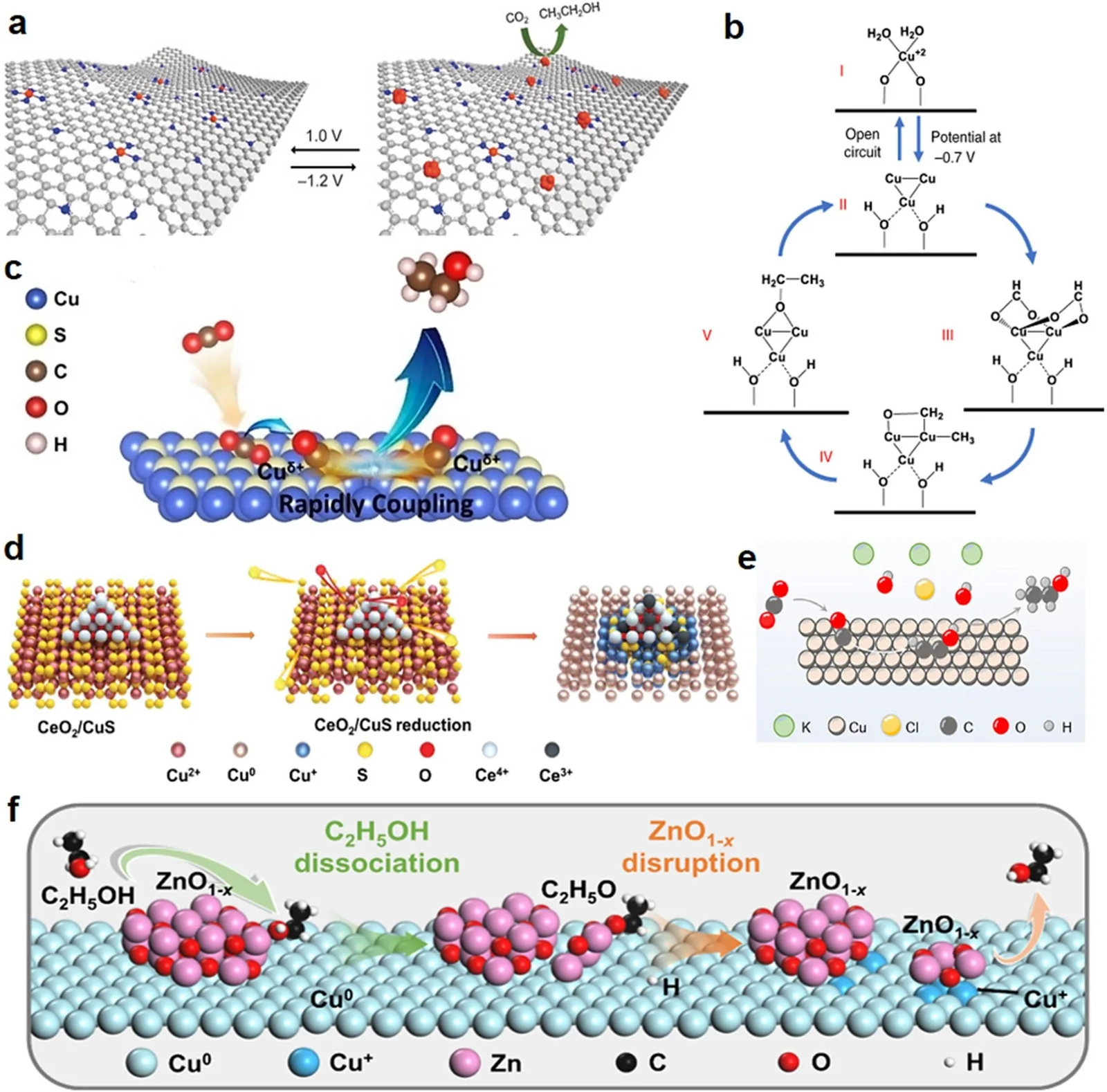
**a** Schematic diagram of the reversible metal site reconstruction mechanism of Cu_0.5_NC during the CO_2_ reduction reaction [75]. Copyright 2019, Wiley–VCH. **b** Hypothesized reaction mechanism suggested by the operando measurements [28]. Copyright 2020, Nature Publishing Group. **c** Schematic diagram of the ethanol production mechanism via eCO_2_RR using Cu_2_S_1−x_ electrocatalyst [76]. Copyright 2022, Wiley–VCH. **d** Schematic representation of the reconstitution process of CeO_2_/CuS [23]. Copyright 2023, Wiley–VCH. **e** Schematic diagram of the mechanism for ethanol production via CO_2_ reduction using Cu_2_(OH)_2_F [25]. Copyright 2025, Wiley–VCH. **f** Schematic diagram of the electrocatalyst structural evolution during C_2_H_5_OH treatment [57]. Copyright 2025, Nature Publishing Group

Jae Young Choi et al. [74] synthesized Cu-SACS-N-CQDs electrocatalysts, in which Cu exists in atomic size and Cu atoms have a unique localized atomic structure and good dispersion on N-CQDs. Subsequently, the eCO_2_RR reaction was carried out in 0.1 M KHCO_3_ aqueous solution, and during CO_2_ electrolysis, it was found that the ligands of neighboring metal Cu–Cu atoms generated in situ by isolated single Cu atoms in the electrocatalyst were real catalytically active sites, which led to the ethanol exhibiting high selectivity. In addition, Marc Fontecave’s group [75] also prepared a Cu single-atom electrocatalyst with CuN_4_ coordination environment by dispersing Cu atoms in a nitrogen-doped conductive carbon matrix, which transformed Cu from its presence in the form of dispersed single atoms in the electrocatalyst to its presence in the form of nanoparticles during electrochemical reconstruction. It was also demonstrated by a series of in situ characterizations that these nanoparticles were the catalytically active substances for the conversion of CO_2_-to-ethanol (Fig. 5a). Similarly, Liu and his colleagues [28] also used the Cu–Li mixed method to prepare a single-atom carbon-supported Cu electrocatalyst. After an electrochemical process, the coordination environment of Cu changed from CuO to Cu–Cu, forming ultrasmall Cu clusters. Under the reaction conditions, there is a dynamic reversible transition between Cu single atom and Cu cluster active center. Upon application of an electric field, the Cu clusters were attached by surface hydroxyl groups, which acted as transient active sites for CO_2_ binding and thus promoted ethanol production. In the absence of an electric field, on the other hand, Cu clusters are very unstable and are easily oxidized by weak oxidants and reduced to Cu single atoms, completing the catalytic cycle (Fig. 5b).

What all these examples demonstrate is that Cu undergoes a dynamic reconfiguration behavior during electrocatalytic CO_2_ reduction, transforming from monoatomic Cu sites with no catalytic activity to small nanoparticles and clusters with significant catalytic activity for ethanol or forming new coordination environments.

In addition to this, with the updating and development of modern in situ characterization techniques, researchers have found that Cu-based electrocatalysts undergo surface remodeling in alkaline electrolytes (KHCO_3_, KOH) accompanied by an elevated oxidation state, which ultimately generates a more catalytically active Cu^+^ species that effectively catalyzes the reduction of CO_2_-to-ethanol [27, 77, 78].

The CeO_2_/CuS nanomaterials designed and constructed by Xi et al. [23] exhibited high selectivity for ethanol during the electrochemical reduction of CO_2_. After in situ characterization, it was demonstrated that the presence of CeO_2_ was able to inhibit the entire self-reduction of CuS, resulting in stable active Cu^0^/Cu^+^ species. At the same time, the remaining S atoms act as electron modulators adsorbed on the Cu surface to promote the conversion of CO_2_ to CO. Thanks to the adaptive structural evolution of this dual modulation effect, the active Cu^+^ site significantly lowers the energy barrier of the C–C coupling and improves the ethanol selectivity (Fig. 5d). Moreover, Zhang’s group [76] also electrochemically prepared a Cu_2_S hollow nanocube with a large number of sulfur vacancies (Cu_2_S_1−x_ HN), which has an ultralow overpotential for the reduction of CO_2_-to-ethanol at 0.19 V. The electron-donating Cu^δ+^ species originating from the S vacancies were shown by attenuated total reflectance Fourier transform infrared spectroscopy (ATR-FTIR) as well as DFT theoretical calculations to play a key role in ethanol formation by lowering the reaction potential of the C–C coupling step as well as facilitating *CO dimerization (Fig. 5c). Additionally, Fan’s research group [25] proposed a strategy using a mixed anion electrolyte (KOH + KCl) to regulate CO_2_ reduction reaction products, significantly enhancing the selectivity and energy efficiency of electrocatalytic CO_2_ reduction to ethanol. In situ XANES further demonstrated that copper exhibits mixed metallic and oxidized states during the eCO_2_RR process (Fig. 5e). Li and colleagues [57] reported a hydrogen-ethanol pretreatment strategy to obtain copper nanoparticles covered by highly dispersed and disordered ZnO_1−x_ clusters. In situ X-ray spectroscopy and computational studies revealed a volcano relationship between the Cu^+^ ratio in copper species and ethanol FE. The optimal Cu^+^ density not only favors *OCCOH coupling but also optimizes the adsorption energy of *CH_2_CH_2_O on the ethanol electrosynthesis electrocatalyst (Fig. 5f).

In summary, the Cu active site usually undergoes reversible or irreversible reconfiguration behavior with the electrochemical process during CO_2_ reduction, which is crucial for the productions of ethanol and even longer carbon chain products. Therefore, exploring the dynamic mechanism of the material during the reaction process will help us to study its reaction mechanism more deeply at the atomic level in the future, thus promoting the development of eCO_2_RR.

### Mechanism Exploration Methodology

In the eCO_2_RR process, many in situ characterization techniques and theoretical methodologies are involved to explore the intrinsic mechanism of the reduction reaction [79–81]. An in-depth understanding of the reaction mechanism of eCO_2_RR will not only help us to design more efficient electrocatalysts, but also improve the stability and selectivity of the reduction reaction, which will promote the goal of carbon neutrality. Firstly, by deeply investigating the reaction pathways and kinetic processes of eCO_2_RR, the design and optimization of electrocatalysts can be guided, thus improving catalytic efficiency and selectivity. For example, the structural evolution and reaction mechanism of electrocatalysts in the in situ state can be investigated using synchrotron radiation technology [82]. In addition, the electronic structure information on the electrocatalyst surface revealed by first-principles calculations can facilitate the rational design of electrocatalysts and the optimization of electrochemical reaction systems. Secondly, the mechanism investigation can help to improve the stability and selectivity of the CO_2_ reduction reaction [83]. For instance, eCO_2_RR in acidic medium can study the influence of cation effect on the electrocatalytic reaction, and the HER can be inhibited and the activity of eCO_2_RR can be enhanced by modulating the cations in the electrolyte. Finally, mechanism investigation is fundamental to promote the development of carbon–neutral technology [84]. Through a deeper understanding of the mechanism of eCO_2_RR, more efficient electrocatalysts can be designed to improve the efficiency and selectivity of CO_2_ conversion, reduce the dependence on fossil fuels, and promote the development of sustainable energy. And the main techniques reported so far are in situ infrared spectroscopy, in situ Raman spectroscopy, in situ synchrotron radiation, and density functional theory (DFT) calculations used to explore the reaction mechanism of eCO_2_RR [85].

#### In Situ* Infrared Spectroscopy*

For mechanistic studies, in situ infrared spectroscopy offers real-time monitoring of electrocatalyst surface dynamics during CO_2_ reduction. By identifying adsorption peaks and transient intermediates, it elucidates active sites and reaction pathways, providing a foundation for improving eCO_2_RR performance through rational electrocatalyst design [86].

**Fig. 6:**
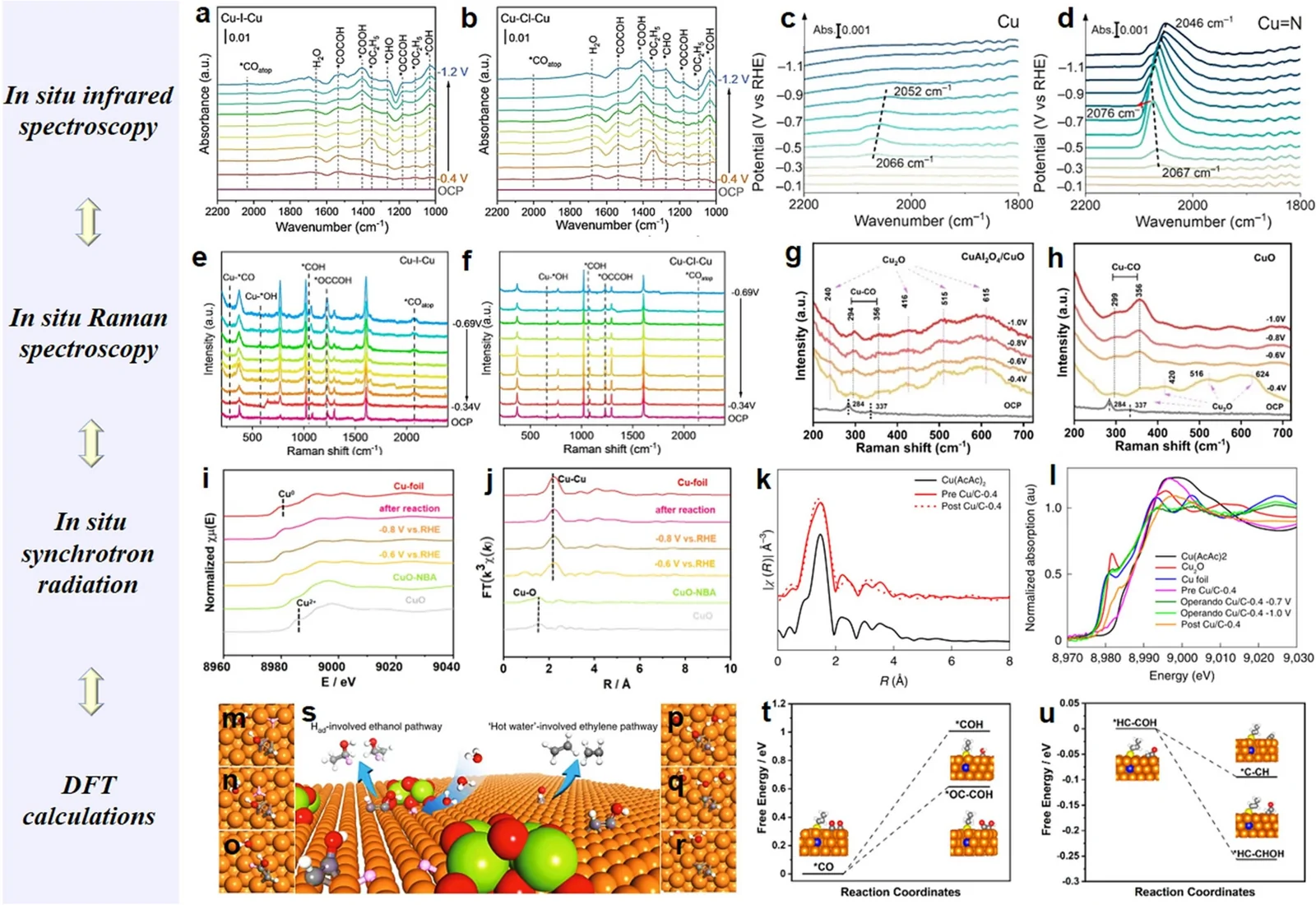
In situ ATR-SEIRAS spectra of **a** Cu–I–Cu and **b** Cu–Cl–Cu dual sites in a potential window from − 0.4 to − 1.2 V [73]. Copyright 2024, Wiley–VCH. In situ IR spectra on the **c** Cu and **d** Cu=N electrocatalysts in a potential window from − 0.1 to − 1.2 V vs RHE [64] Copyright 2024, American Chemical Society. In situ Raman spectra for adsorbed intermediates on **e** Cu–I–Cu and **f** Cu–Cl–Cu [73]. Copyright 2024, Wiley–VCH. In situ Raman spectra of **g** CuAl_2_O_4_/CuO and **h** CuO during eCO_2_RR at OCP and different applied potentials at a laser excitation wavelength of 532 nm [89]. Copyright 2023, Wiley–VCH. **i** The in situ XANES spectra at the Cu K-edge for Cu-NBA at different potential during eCO_2_RR. **j** The corresponding Fourier transforms FT(*k*^3^*w*(*k*)) for Cu-NBA at different potential during eCO_2_RR [94]. Copyright 2024, Wiley–VCH. **k** Fourier transform of *k*^2^-weighted *R* space *χ* EXAFS data of the postmortem electrocatalysts of Cu/C-0.4 after 16 h chronoamperometry measurement and Cu(AcAc)_2_ as a reference. **l** In situ Cu k-edge XANES spectra of pre-Cu/C-0.4, Cu/C-0.4 at − 0.7 V vs RHE and − 1.0 V vs RHE and post-Cu/C-0.4 [28]. Copyright 2020, Nature Publishing Group. Top views of geometries **m** initial state, **n** transition state, and **o** final state of key reaction toward ethanol, and **p** initial state, **q** transition state, and **r** final state of key reaction toward ethylene. Red, white, gray, and orange balls stand for oxygen, hydrogen, carbon, and copper, respectively, while pink balls stand for H_ad_ on Cu [24]. Copyright 2019, Nature Publishing Group. **t** Free energy differences of *COH and *OCCOH formation and the optimized adsorption structures of *CO, *COH, and *OC–COH on CuBr-DDT, respectively. **u** Free energy differences of *C–CH and *HC–CHOH formation and the optimized adsorption structures of *HC–COH, *C–CH, and *HC–CHOH on CuBr-DDT, respectively [32]. Copyright 2021, American Chemical Society

Wang and his research team [73] constructed Cu-Cu double sites with different site distances by halogen coordination, which showed high selectivity for the generation of C_2_ products. They probed the dynamic behavior of the reaction intermediates using in situ electrochemical ATR-SEIRAS spectroscopy. Figure 6a, b reveals that the peaks located at ~ 2050 and ~ 1395 cm ^−1^ correspond to CO and COOH intermediates, respectively, indicating the activation and initial reduction steps of CO_2_.The peaks at 1535 and 1190 cm^−1^ displacements belong to COCOH and OCCOH intermediates, revealing the C–C coupling process, whereas the peaks at about 1035 and 1270 cm^−1^ displacements denote COH and CHO intermediates, showing the pathway of CO_2_ reduction to ethanol. Thus, the in situ infrared spectroscopy in this work reveals the effect of different Cu-X-Cu double sites on the dynamic behavior of CO_2_ reduction reaction intermediates.

In contrast, Zheng et al. [64] utilized a nitroxide functionalization method to create an electronically delocalized state on the surface of a Cu electrocatalyst formed by a Cu=N double bond and an aryl sulfonyl nitroso conjugated *π*-bond. This surface nitroso functionalization facilitates electron delocalization from the Cu atom to the aryl sulfonyl nitroso molecule, which can accelerate the cleavage of the Cu–O bond, thus favoring the pathway to ethanol production. They, in turn, compared the surface *CO coverage of the copper electrocatalysts using in situ attenuated total reflection surface-enhanced infrared absorption spectroscopy, as shown in Fig. 6c, d, the three electrocatalysts showed peaks between 2100 and 2000 cm^−1^ attributed to linearly bonded CO_L_. The Cu=N electrocatalysts exhibited stronger CO_L_ peaks over a wider voltage window compared to the other two control samples, suggesting that the Cu=N electrocatalysts have a higher *CO surface coverage, which increases the probability of C–C coupling and promotes ethanol production. In this work, they used in situ infrared spectroscopy to see the coverage of CO intermediate, which are crucial in the pathway to ethanol generation, and thus to determine the effect of intermediate coverage on the CO_2_ reduction reaction.

Accordingly, in situ infrared spectroscopy plays a vital role in studying the dynamic behavior of intermediates, exploring the microstructure and reactive sites of electrocatalysts, as well as exploring the reaction mechanism, and is an irreplaceable technique for in situ characterization.

#### In Situ* Raman Spectroscopy*

In situ Raman spectroscopy is also one of the most common methods of mechanism investigation, which is able to characterize the subtle changes in electrochemical reactions, and has been widely applied in several fields [87]. It can monitor the changes of the electrocatalyst in the reaction process in real time, directly observe the intermediate products and the reaction process during the electrocatalytic reaction, and also obtain dynamic information about the molecular structure and vibration of the sample [88]. Therefore, it is of great significance for the study of electrocatalyst activity, electrode reaction process, and reaction mechanism.

As mentioned in the previous subsection, the work of Wang and his research team [73], who also used in situ Raman in their experiments, probed the CO_2_ reduction reaction path of the Cu–X–Cu double site. Figure 6e, f shows that Cu–I–Cu has an obvious Cu–CO signal in the range of 200–500 cm^−1^, showing its stronger CO_2_ activation ability. And the peak at 2090 cm^−1^ displacement is a CO vibrational peak, which is closely related to the C–C coupling, indicating that Cu–I–Cu has a superior C–C coupling ability. In addition, Cu–Cl–Cu has a stronger Cu–*OH peak at 280 cm^−1^, indicating its stronger water activation ability, which is favorable for ethanol generation. Thus, this work reveals that short-range Cu-Cu sites favor CO_2_ adsorption and ethanol generation by in situ Raman technique, while long-range Cu–Cu sites are more favorable for C–C coupling and C_2_ product generation.

Not only that, Xu Xiang’s team [89] prepared a layered precursor-derived CuAl_2_O_4_/CuO electrocatalyst for efficient ethanol electroproduction. They used in situ Raman spectroscopy to monitor the catalytic behavior of the intermediate in the eCO_2_RR process and determined the real catalytic site of the reaction. As shown in Fig. 6g, h, the peaks of CuO were displayed under OCP, but the peaks of CuO disappeared after applying a potential to the electrocatalyst. In addition, new Raman peaks with different phonon modes belonging to Cu_2_O species appeared for CuAl_2_O_4_/CuO at an applied potential of − 0.4 V. And the Cu^+^ species in CuAl_2_O_4_/CuO can be maintained stable within a wide potential window from − 0.4 to − 1.0 V. And the signal of Cu^+^ species in CuO disappeared at − 0.6 V, reflecting the instability of Cu^+^ species in CuO during the eCO_2_RR process. The results indicated that CuAl_2_O_4_ enhanced the stability of Cu^+^ species in the main sample and promoted the generation of ethanol at the active Cu^+^ sites. Besides, both electrocatalysts showed adsorbed *CO peaks at about 299 and 356 cm^−1^ belonging to Cu–CO frustrated rotation and Cu–CO stretching. Thus, CuAl_2_O_4_ in the electrocatalysts played a critical role in stabilizing the active Cu^+^ species.

In conclusion, in situ Raman technology is very pivotal in the application of electrocatalysis, which can monitor the internal structure, chemical composition, and dynamic changes of the material in real time, helping us to deeply understand the mechanism of electrochemical reaction and the law of change of performance, and providing powerful support for the design and optimization of materials.

#### In Situ* Synchrotron Radiation*

In situ synchrotron radiation technology, with its high brightness, high-energy resolution, broad spectral coverage, and ultrafast temporal resolution capabilities, has emerged as another powerful tool for unraveling the mechanistic challenges of eCO_2_RR [90, 91]. It enables simultaneous acquisition of information on the electronic structure, crystal structure evolution, and formation/transformation of surface intermediate species under reaction conditions, revealing the reaction pathways and regulatory mechanisms of eCO_2_RR at the atomic/molecular scale [92, 93]. This not only provides direct evidence for understanding the dynamic evolution of catalytic active sites but also facilitates the precise design of eCO_2_RR electrocatalysts with high activity, selectivity, and stability.

Han et al. [94] synthesized Cu nanoparticles modified with amine molecules featuring varying alkyl chain lengths via a two-step method. They employed in situ synchrotron radiation experiments to confirm the active sites involved in this reaction process. As shown in Fig. 6i, under negative potential, the X-ray absorption near-edge structure (XANES) spectrum of Cu–NBA exhibits characteristics similar to Cu foil, indicating that metallic Cu predominates in Cu-NBA during the eCO_2_RR process. The * k*^3^-weighted Fourier transform (FT) extended X-ray absorption fine structure (EXAFS) spectrum reveals that only the Cu–Cu bond peak of Cu–NBA is observed during the eCO_2_RR process (Fig. 6j). These results indicate that in the CO_2_ reduction reaction, CuO is completely reduced to Cu, with metallic Cu serving as the core of the electrocatalyst.

Additionally, Xu’s group [28] prepared a single-atom carbon-supported copper electrocatalyst (Cu/C-0.4) using a Cu–Li hybrid approach. They investigated the EXAFS of Cu/C-0.4 before and after chronoamperometry measurements, revealing an almost fully coordinated CuO shell around the Cu/C electrocatalyst in both states. This indicates that Cu remains atomically dispersed even after prolonged electrocatalysis (Fig. 6k). Immediately after applying − 0.7 V, reduction from ionic Cu to metallic Cu was observed (Fig. 6l). EXAFS analysis further revealed that at the electrochemical potential, the coordination environment of Cu shifted from CuO to predominantly Cu–Cu bonds with coordination numbers of 2 (± 0.9) or 3 (± 1.2), indicating the formation of ultramicroscopic Cu species, Cun, where *n* = 3 or 4. XAS studies revealed a crucial phenomenon in the electrocatalyst: a dynamic reversible transformation between SA and Cun active sites under reaction conditions.

In summary, in situ synchrotron radiation enables dynamic tracking at the atomic/molecular scale under actual reaction conditions, establishing for the first time a direct structure–function relationship linking “reaction environment-microstructure-macroscopic properties.” For instance, in the field of eCO_2_RR, it allows real-time capture of valence state evolution at active sites and the formation or transformation pathways of intermediates, making it one of the most essential techniques in contemporary advanced characterization.

#### Density Functional Theory (DFT) Calculations

Unlike the first two test methods, density functional theory calculations are a quantum mechanical theoretical approach to study the electronic structure and physicochemical properties of materials in a multielectron system [14]. Its main idea is to take the total energy of a multielectron system as a function of the electron density and to solve the ground state properties of the system by minimizing the energy function [95]. DFT calculations can be applied in the eCO_2_RR system to understand and predict the mechanism of catalytic reactions, help researchers understand the electronic behavior of electrochemical reactions through structure optimization and surface free energy calculations, and predict the energy path of electrochemical CO_2_ reduction reactions [96].

Edward H. Sargent’s group [24] utilized a complementary approach to doping the electrocatalyst surface with hydroxides and oxides to regulate the adsorption of hydrogen on Cu. They elucidated the mechanism of this approach through DFT calculations, as shown in Fig. 6m-s, where the removal of OH from *HCCOH in the ethylene pathway to form *CCH is due to the involvement of surface water molecules in the removal of OH: In the transition state, the O-C bond between the hydroxyl group and *CCH dissociates in the presence of surface water. In the final state, OH is stabilized by water and forms *CCH. Thus, surface water molecules play a crucial role in the ethylene pathway. And in the ethanol pathway, Had attacks *HCCOH and *HCCOH is hydrogenated to *HCCHOH, which is a key intermediate in the production of ethanol. And Had is only involved in the conversion reaction to ethanol. So, increasing the coverage of Had will enhance the efficiency of ethanol generation. In summary, this doping can promote the dissociation of water and change the adsorption energy of hydrogen on Cu, which in turn changes the selectivity of the product.

Wu et al. [32] constructed a dodecanethiol-modified CuBr electrocatalyst based on dodecanethiol modification that can generate stable bromine-doped copper thiol interfaces in situ, which exhibits excellent selectivity for ethanol generation. As shown in Fig. 6t, u density functional theory simulations of the key reaction steps and the energy distribution of the optimized atomic structure during this reaction, it can be seen that the adsorption of DDT inhibits the selectivity of H_2_ and CH_4_. The doping of Br species in Cu stabilizes the higher-valent Cu species, which improves the ethanol selectivity and even the selectivity of the C_2+_ product. Thus, DFT calculations show that the improvement of electrocatalytic activity is related to the modulation of the adsorption energy of key intermediates on the bromine-doped thiol-Cu interfaces, and the present work also demonstrates the great value of the research on the use of hybridized metal–molecule interfaces to improve the electrocatalytic CO_2_ performance.

In summary, DFT calculations have become an indispensable part of electrochemical mechanistic studies and have also been widely applied in various fields such as the study of molecular structures and electronic properties of materials.

## Design of Electrocatalysts for Electrochemical CO_2_ Reduction to Ethanol

Currently, various metals are used as electrocatalysts in eCO_2_RR to produce valuable compounds. A detailed summary of the different types of electrocatalysts for ethanol production from eCO_2_RR is presented in this chapter.

### Copper-Based Electrocatalysts

**Fig. 7:**
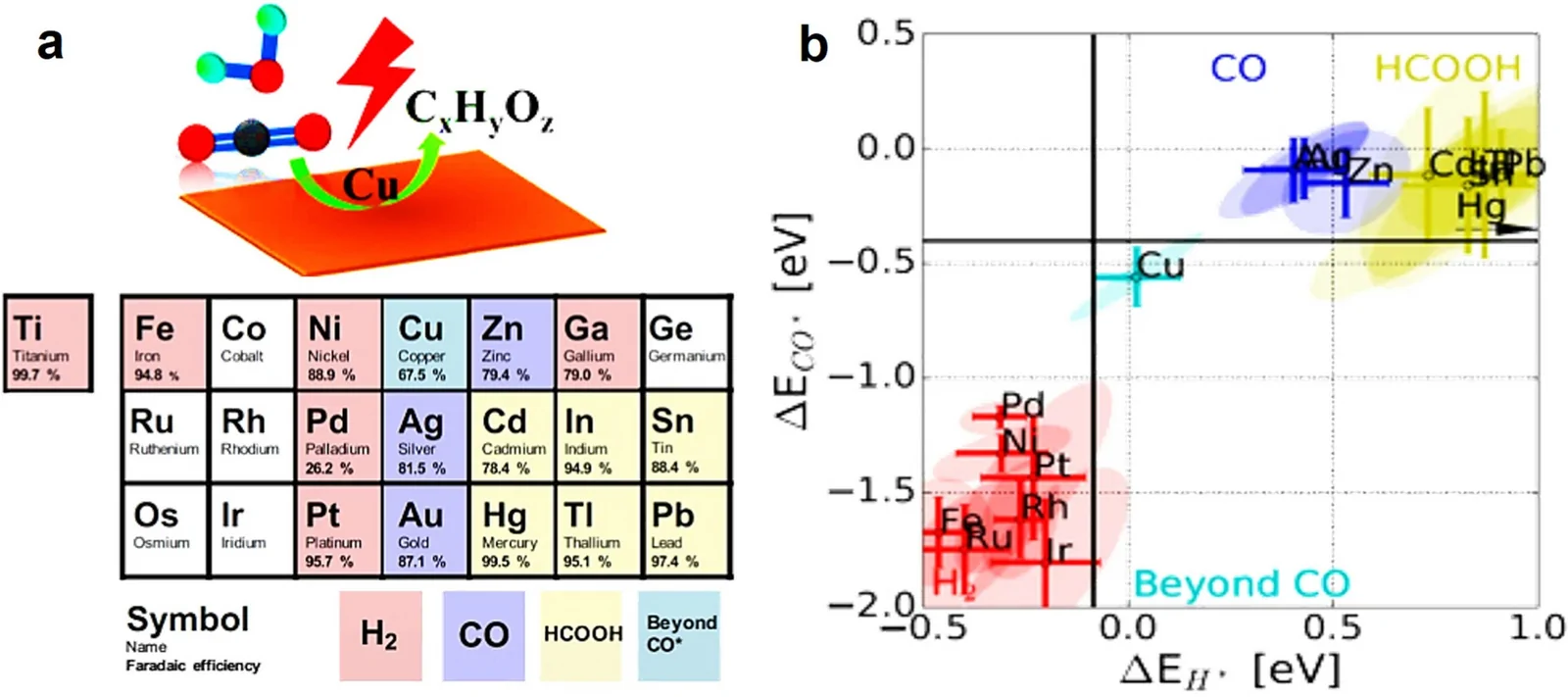
**a** Classification of the main products of eCO_2_RR metal electrocatalysts. **b** CO_2_ reduction metal classification [97]. Copyright 2017, John Wiley and Sons

For different metal elements, as shown in Fig. 7, the adsorption energy (ΔE_H*_) of elemental copper for the intermediate H* is weak, while the adsorption energy (ΔE_CO*_) for the intermediate CO* is moderate, and therefore, copper is able to attenuate the hydrogen evolution reaction (HER) effectively to some extent, and to enhance the C–C coupling or the hydrogenation process of CO* intermediates [97, 98]. Due to this property, copper plays a significant part in converting CO_2_ to C_2+_ products and is one of the very few metal electrocatalysts capable of converting CO_2_-to-ethanol [99–101]. At present, reports on eCO_2_RR ethanol production are still dominated by Cu-based electrocatalysts. This subsection focuses on the research progress of Cu-based electrocatalysts such as Cu–N-doped carbon materials, oxide-derived copper, and copper alloys.

#### Copper-Based Carbon Supported Electrocatalysts

Carbon-based materials are widely used as substrate materials owing to their large specific surface area, excellent electrical conductivity, strong adsorption capacity, and other physical and chemical properties [102, 103]. Nitrogen doping can enhance the surface polarity of carbon-based materials, improve surface wettability, reduce the energy barrier during ion adsorption and desorption, and modify the surface electronic structure of carbon atoms, which is an effective means to improve the physicochemical properties of carbon-based materials [104]. Research has demonstrated that the combination of copper and N-doped carbon material producing CO can produce highly selective ethanol through the series catalytic mechanism [105–107]. By reducing the size of copper particles to the atomic level and anchoring to the N-doped carbon material, the atomically dispersed Cu–N–C material is adjusted in terms of electronic structure, geometric structure, and energy level structure, so that the synthesized electrocatalyst has excellent eCO_2_RR performance [108, 109].

**Fig. 8:**
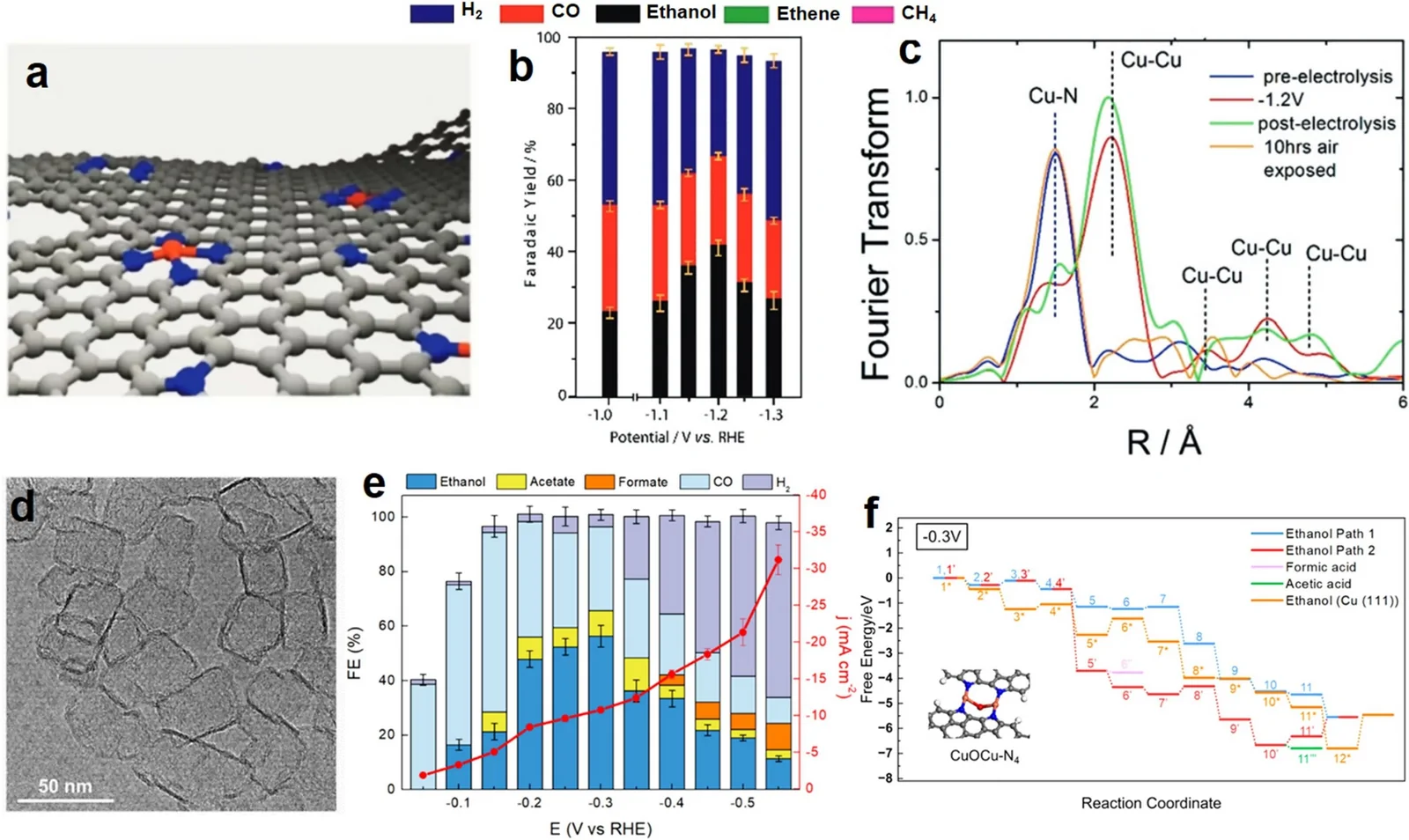
**a** Schematic representation of Cu_0.5_NC. **b** Faradaic efficiency of Cu_0.5_NC in 0.1 M CsHCO_3_ aqueous solution at 2.5 mL min^−1^ CO_2_ flow rate under various applied potentials. **c** Fourier transform of the experimental EXAFS spectra of Cu_0.5_NC under no potential applied (blue line), Cu_0.5_NC during electrolysis at − 1.2 V vs. RHE (red line), after electrolysis under no potential applied (green line) and Cu_0.5_NC after electrolysis at − 1.2 V vs. RHE then sample exposed to air for 10 h (orange line) [75]. Copyright 2019, Wiley–VCH. **d** TEM image of Cu − 1/hNCNC. **e** FE, j, and product distribution for Cu − 1/hNCNC at different polarization potentials. The data were averaged over three repeated measurements. The error bars are marked for ethanol and total products. **f** Free energy diagrams of eCO_2_RR-to-ethanol (Path 1 and Path 2), formic acid, and acetic acid [110]. Copyright 2024, American Chemical Society

Fontecave et al. [75] dispersed copper atoms in nitrogen-doped conductive carbon matrix and prepared a single copper atom electrocatalyst with CuN_4_ coordination environment (Fig. 8a) through a simple pyrolytic synthesis pathway. Experiments show that the isolated CuN_4_ site can be used as a precursor of highly active electrocatalyst for CO_2_ electroreduction of ethanol, and ethanol can be generated as the only liquid product, and the ethanol Faraday yield is as high as 55% at − 1.2 V vs. RHE (Fig. 8b). Moreover, FT-EXAFS spectra clearly show that Cu was essentially present as nanoparticles in the electrocatalyst after prolonged electrolysis at − 1.2 V vs. RHE (Fig. 8c). In situ characterization proved that these nanoparticles were catalytic active substances for the conversion of CO_2_-to-ethanol.

In addition, Hu et al. [110] used Cu_2_O as the precursor and unique hierarchical nitrogen-doped carbon nanocages (hNCNCs) as the carrier to construct a electrocatalyst with a novel CuOCu–N_4_ binuclear site and Cu–N_4_ unit point coexistence through micropore capture and nitrogen anchoring. Meanwhile, the CuOCu–N_4_ binuclear site retains about 3.032 A Cu··Cu spacing in the Cu_2_O precursor (Fig. 8d). The electrocatalyst showed remarkable activity in CO_2_ ethanol production, with the overpotential of ethanol as low as 0.19 V and FE as high as 56.3% at − 0.3 V (Fig. 8e). DFT calculation illustrates that the CuOCu–N_4_ binuclear site spontaneously promotes C–C coupling, whereas the coexisting Cu–N_4_ unit site and hNCNCs carriers can provide additional CO species, facilitating tandem electrocatalytic ethanol production (Fig. 8f). This work provides a new line of thought to gain insight into Cu dinuclear site electrocatalysts for ethanol production from eCO_2_RR.

It is indisputable that understanding the local structure and electronic state of the active site is crucial to understanding the catalytic mechanism and reaction pathway involved in the conversion of CO_2_ to alcohol. Nevertheless, the current use of multidimensional in situ monitoring of the reaction system may also allow for the exploration of the mechanism of the effect of the atomic constitutive relationships of the electrocatalyst on the activity and selectivity.

**Fig. 9:**
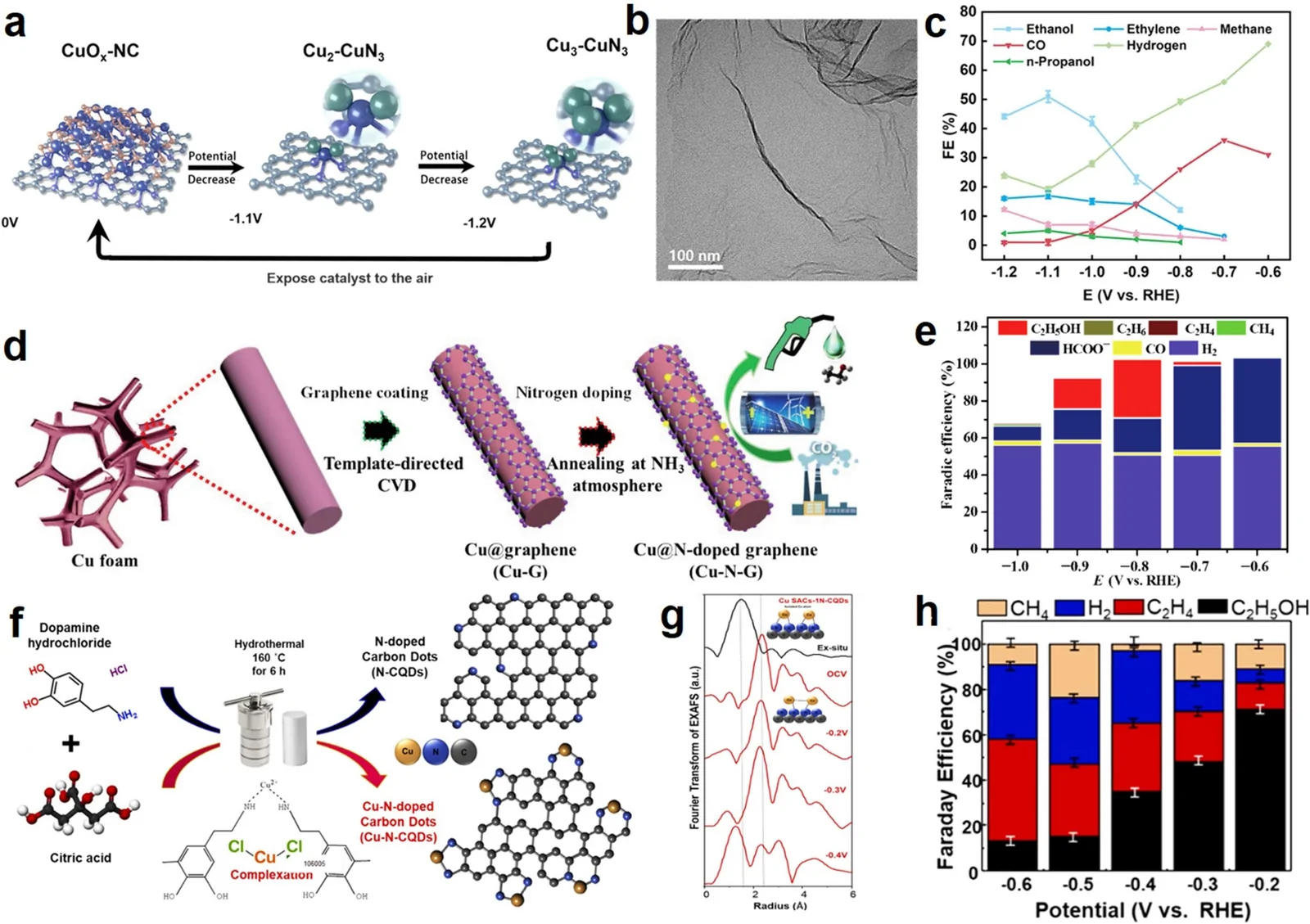
**a** Proposed scheme for the reversible formation of the catalytically active Cu_n_-CuN_3_ cluster based on operando XAS and Quasi-in situ XPS analysis (rufous, O; gray, C; purple, N; blue, Cu bond to both N and Cu; green, Cu just bond to Cu). **b** TEM image of Cu/N_0.14_C. **c** FE of each product of Cu/N_0.14_C [111]. Copyright 2022, Nature Publishing Group. **d** Schematic preparation process of Cu@N-doped graphene electrocatalyst for electrocatalytic CO_2_ reduction. **e** Faradic efficiency of the products at selected potentials for Cu–N–G electrocatalysts [114]. Copyright 2022, Nano Research. **f** Overall synthesis process for the Cu-SACs-N-CQDs electrocatalyst synthesis process. **g** Operando XAS characterization of the Cu-SACs-1N-CQDs. **h** Faradaic efficiency at different potential with 0.125 dopamine (Cu-SACs-1N-CQDs) [74]. Copyright 2024, Elsevier B.V

Wang et al. [111] synthesized a CuO cluster loaded on nitrogen-doped carbon nanosheets as an electrocatalyst (Fig. 9b). By adjusting the nitrogen content on the carrier to regulate the electron transfer and interaction between the Cu species and the carrier, the optimal electrocatalyst can demonstrate up to 73% Faradaic efficiency of the C_2+_ product at the potential of − 1.1 V vs. RHE, including 51% ethanol Faradaic efficiency (Fig. 9c). And the electrocatalyst showed outstanding long-term stability of CO_2_ electroreduction within 10 h. The reversible potential-dependent transition from the dispersed CuO cluster to the Cu_2_–CuN_3_ cluster as the best site was determined by Operando XAS, XANES simulations, and quasi-in situ XPS analysis (Fig. 9a). When voltage is applied, CuO clusters change into metastable Cu_2_-CuN_3_ clusters in order to take part in the catalytic reaction. When voltage is removed, the electrocatalyst returns to its stable state of CuO clusters. The charge distribution between Cu atoms and N-doped carbon surfaces is adjusted to maintain the high activity and outstanding stability of Cun clusters. Cu_2_-CuN_3_ clusters are found to have charge asymmetry sites by combining Operando FTIR and DFT theoretical calculation; CH_3_* adsorption strengthens these sites, which is advantageous to the synthesis of highly efficient asymmetric ethanol.

In recent years, graphene, as a novel form of two-dimensional carbon atomic material, has attracted wide attention in eCO_2_RR to C_2+_ products due to its special structure, outstanding thermal conductivity and mechanical properties [112, 113]. In order to specifically increase ethanol selectivity in eCO_2_RR, researcher Dejin Zang adopted a template-oriented CVD strategy to create a thin layer of N-doped graphene (Cu–N–G) on the surface of copper foam (Fig. 9d) [114]. The ethanol selectivity is increased to 33.1% by the total Faradaic efficiency of Cu–N–G at − 0.8 V vs. RHE, in comparison with the undetectable ethanol selectivity of pure Cu and Cu–G (Fig. 9e). The results of experiments and DFT calculations show that the interconnect graphene coating can be used as a fast charge transfer channel while also providing a limited nanospace for mass transfer. On the other hand, the limiting effect of the Cu–N-G interface not just provides high adsorbed hydrogen coverage, but stabilizes the critical *HCCHOH intermediates in the direction of the ethanol route. It is anticipated that this work’s interface enhancement method will encourage the development of Cu-based eCO_2_RR electrocatalysts so as to produce C_2+_ products.

In general, there are two common strategies for the synthesis of Cu-SACs atoms catalysts on N-doped carbon support: top-down and bottom-up [115]. Top-down synthesis techniques involve dispersing Cu-SACs atoms onto already-existing carbon carriers (graphene or CNTs) or synthetic carbon [116]. The bottom-up strategy involves mixing Cu metal with organic precursors and then forming a carbon matrix in a high-temperature carbonization process, usually above 800 °C, in which Cu metal atoms are embedded in the carbon [74, 117]. However, due to the shortcomings of complex synthesis, high cost, high-energy consumption, and low yield, the SACs synthesis method has low selectivity, low efficiency, and low stability for ethanol production [118]. Consequently, it is necessary to develop single-atomic Cu electrocatalysts with straightforward method, low temperature, and high selectivity for C_2_H_5_OH.

Based on this, Baik et al. [74] proposed a simple metal-amine (Cu-dopamine) complex and low-temperature hydrothermal method to prepare Cu-SACs-N-CQDs electrocatalyst using dopamine as the N-anchoring source, which contains isolated copper atoms dispersed on the nitrogen-doped carbon point (2–6 nm) support (Fig. 9f). In addition, copper metal coordination and local environmental loading can be regulated by changing the dopamine content. Furthermore, the atomic dispersed Cu-SACs-N-CQDs solution is sufficiently stable for more than six months to show its stability. At the same time, the optimized Cu-SACs-N-CQDs electrocatalyst can produce ethanol at − 0.2 V vs. RHE with a Faradaic efficiency of 70% and a 50-h operating stability (Fig. 9h). In situ EXAFS confirms that isolated Cu atoms are evenly distributed on N-doped carbon points. Isolated Cu atoms evolve into active species during CO_2_ reduction from adjacent Cu–Cu atom coordination, which is helpful for the synthesis of ethanol (Fig. 9g). This work shows that Cu-SACs has great potential for eCO_2_RR to C_2_H_5_OH at low overpotential and for switching reaction paths from C_2_H_5_OH to C_2_H_4_.

In conclusion, by adjusting the electronic structure, geometric structure, and energy level structure of Cu–N–C material, it can have excellent ethanol production performance of eCO_2_RR.

#### Oxide Derived Copper Electrocatalysts

In the eCO_2_RR process, C_2_H_5_OH and C_2_H_4_ are both 12e^−^ reduction products and share the initial intermediate *HCCOH. Compared with C_2_H_4_, the structure of C_2_H_5_OH is more saturated, and the next intermediate of C_2_H_5_OH is more challenging to stabilize on a pure copper surface [119, 120]. In order to boost the yield of C_2_H_5_OH, it is also an effective strategy to modify Cu with other CO_2_^−^active metals to create Cu-based bimetallic compounds.

**Fig. 10:**
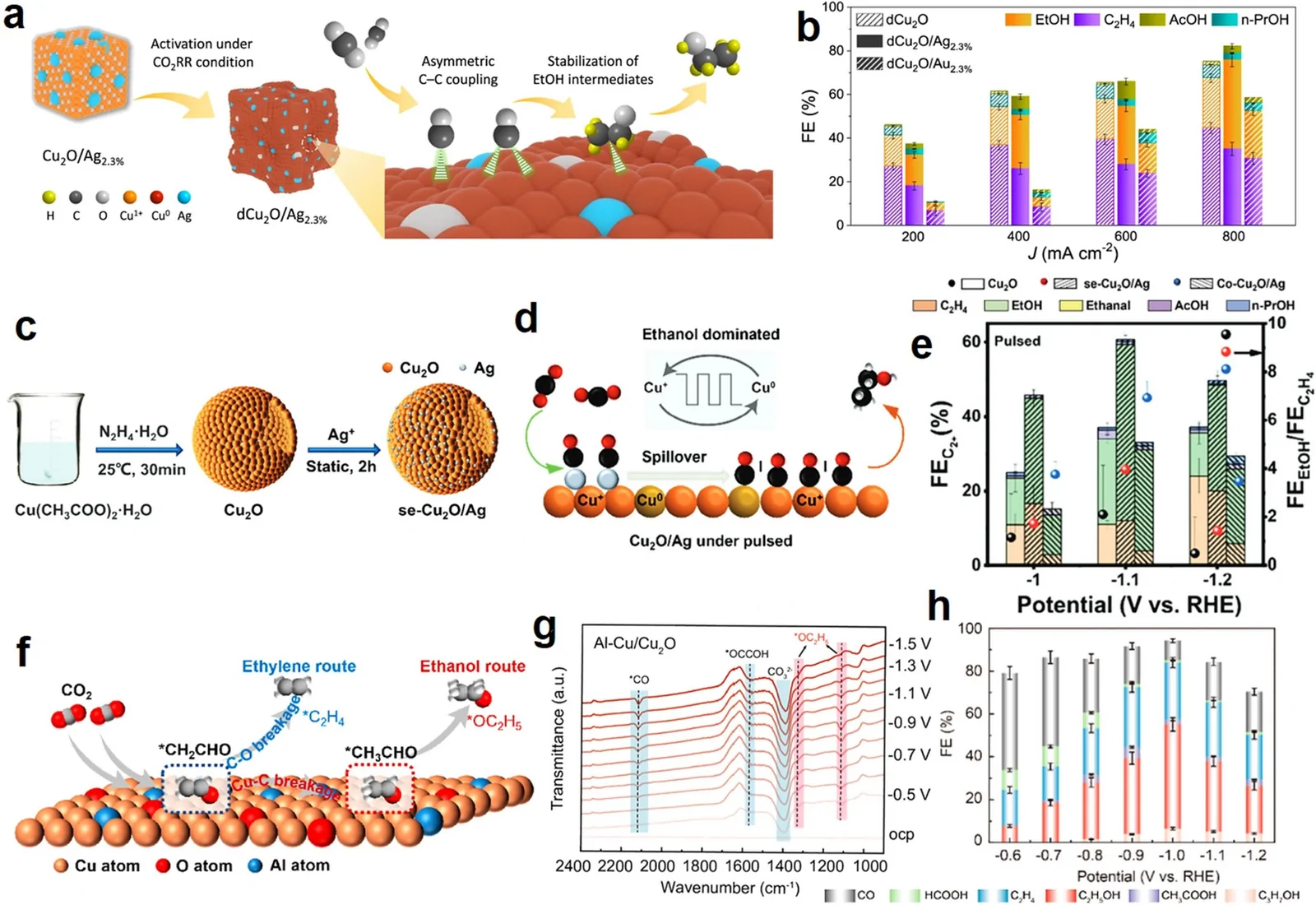
**a** Schematic for boosted EtOH generation over dCu_2_O/Ag_2.3%_. Yellow-color, gray, white, orange, red, and azure spheres in the model represent H, C, O, Cu^1+^, Cu^0^, and Ag atoms, respectively. **b** FE value of C_2+_ products for dCu_2_O, dCu_2_O/Au_2.3%_, and dCu_2_O/Ag_2.3%_ under selected current density [121]. Copyright 2022, Nature Publishing Group. **c** Schematic illustration of se-Cu_2_O/Ag. **d** Schematic of eCO_2_RR process for se-Cu_2_O/Ag under pulsed electrolysis. **e** FE of C_2+_ products (left axis) and FE_EtOH_/FE_C2H4_ (right axis) at various applied potentials inCO_2_-saturated 0.1 M KHCO_3_ under pulsed electrolysis (*E*_*a*_ = + 0.4 V versus RHE, *t*_*a*_ = *t*_*c*_ = 0.5 s) [123]. Copyright 2023, Wiley–VCH. **f** Diagram of the ethanol and ethylene pathways for the eCO_2_RR (to produce SDI and EDI). **g** In situ ATR-FTIRS spectra recorded at different potentials for Al–Cu/Cu_2_O. **h** FEs of various products at different potentials for Al–Cu/Cu_2_O [60]. Copyright 2023, American Chemical Society

Qiao and his colleagues designed and developed a CuAg bimetallic electrocatalyst (dCu_2_O/Ag2.3%), which modified Ag on the surface of Cu_2_O and activated it by electrochemical reduction, effectively regulating the valence state and coordination number of the Cu site on the electrocatalyst’s surface [121]. This also led to the change of the adsorption configuration of the reaction intermediate *CO on the Cu sites during the eCO_2_RR process. *CO coexists in both top adsorption and bridge adsorption on dCu_2_O/Ag2.3% electrocatalyst in comparison with the pure Cu electrocatalyst. The DFT calculation shows that the energy required for the protonation of *CHO or *COH from the bridge adsorption of *CO at Cu site is lower than that for the top adsorption of *CO. Therefore, on the dCu_2_O/Ag2.3% electrocatalyst, the bridge adsorbed *CO tends to preferentially protonate, and then coupling with the top adsorbed *CO, that is, asymmetric *CO–*CHO(*COH) coupling is triggered. This asymmetric C–C coupling has more advantages on the reaction energy barrier than direct dimerization of *CO, and can effectively accelerate the synthesis of C_2+_ products. Meanwhile, the asymmetric C–C coupling leads to the destruction of the originally balanced coordination environment at the Cu site, making it easier to stabilize the ethanol intermediate *OC_2_H_5_ with higher saturation, and guiding the reaction to the ethanol generation path (Fig. 10a). The results of high current catalytic test of CO_2_ in the flow cell show that HER is effectively inhibited under the action of dCu_2_O/Ag2.3% electrocatalyst, and the unfavorable competition between ethanol and ethylene is also improved. The Faradaic efficiency and partial current density of the final product ethanol can reach 40.8% and 326.4 mA cm^−2^, respectively, at − 0.87 V (Fig. 10b). Besides, the electrocatalyst has been further applied to more practical membrane electrodes, and also obtained excellent ethanol selectivity.

It was found that the selectivity of copper oxide to ethanol was significantly improved after reduction because of the presence of Cu^+^ on the electrocatalyst surface [122–124]. At the same time, the theoretical calculation also shows that the presence of both Cu^+^ and Cu^0^ on the surface of the Cu-based electrocatalyst will have a synergistic effect that will significantly enhance the reaction process of CO_2_ activation and CO dimerization, ultimately improving the efficiency and selectivity of eCO_2_RR [125].

In Wu’s work, an efficient electrocatalyst with an enriched Cu_2_O/Ag interface consisting of Cu_2_O hollow nanospheres loaded with Ag nanoparticles (se-Cu_2_O/Ag) was synthesized, and excellent ethanol performance was obtained by pulsed CO_2_ electrolysis (Fig. 10c) [123]. The Faradaic efficiency of C_2_H_5_OH obtained by pulsed CO_2_ electrolysis was significantly increased to 46.3% in a neutral flow electrolytic cell with a partial current density as high as 417 mA cm^−2^, which was significantly superior to the performance of the conventional static electrolytic Cu electrocatalyst (Fig. 10e). In situ spectroscopy and DFT calculations confirmed that the stabilization of the Cu^+^ fraction of se-Cu_2_O/Ag during pulsed electrolysis improved the coverage of *CO. The addition of Ag facilitated the coupling of *CO and *CH, and the reaction potential for the hydrogenation of *HCCOH to ethanol was decreased by stabilized Cu^+^ under pulsed electrolysis (Fig. 10d). This work demonstrates an efficient ethanol production strategy combining electrocatalyst design and pulsed electrolysis, providing some insights into the development of advanced eCO_2_RR technology for the production of high-value products.

In addition, Buxing Han’s Group introduced another method to modulate the adsorption of oxygen-related active species on Cu by adding an oxygenophilic metal, which could also be effective in improving the ethanol selectivity [60]. They doped the Lewis acid metal Al into the Cu-based electrocatalysts, and the prepared Al–Cu/Cu_2_O electrocatalysts exhibited excellent C_2+_ product selectivity in a flow cell, where the FE of C_2+_ alcohols was as high as 55.2%; and the current densities and yields of the C_2+_ alcohol fraction were 354.2 mA cm^−2^ and 1066.8 μ mol cm^−2^ h^−1^ (Fig. 10h). Experimental studies showed that, as a Lewis acid site, Al doping can stabilize the Cu+ fraction, improve the adsorption strength of reactive oxygen species on the Cu surface, stabilize the key intermediate *OC_2_H_5_, and inhibit the C–O bond breaking in the *CH_2_CHO intermediate for the formation of ethylene, which results in a high ethanol selectivity to ethanol (Fig. 10f, g). Also, this approach can be generalized to other Lewis acid metals.

**Fig. 11:**
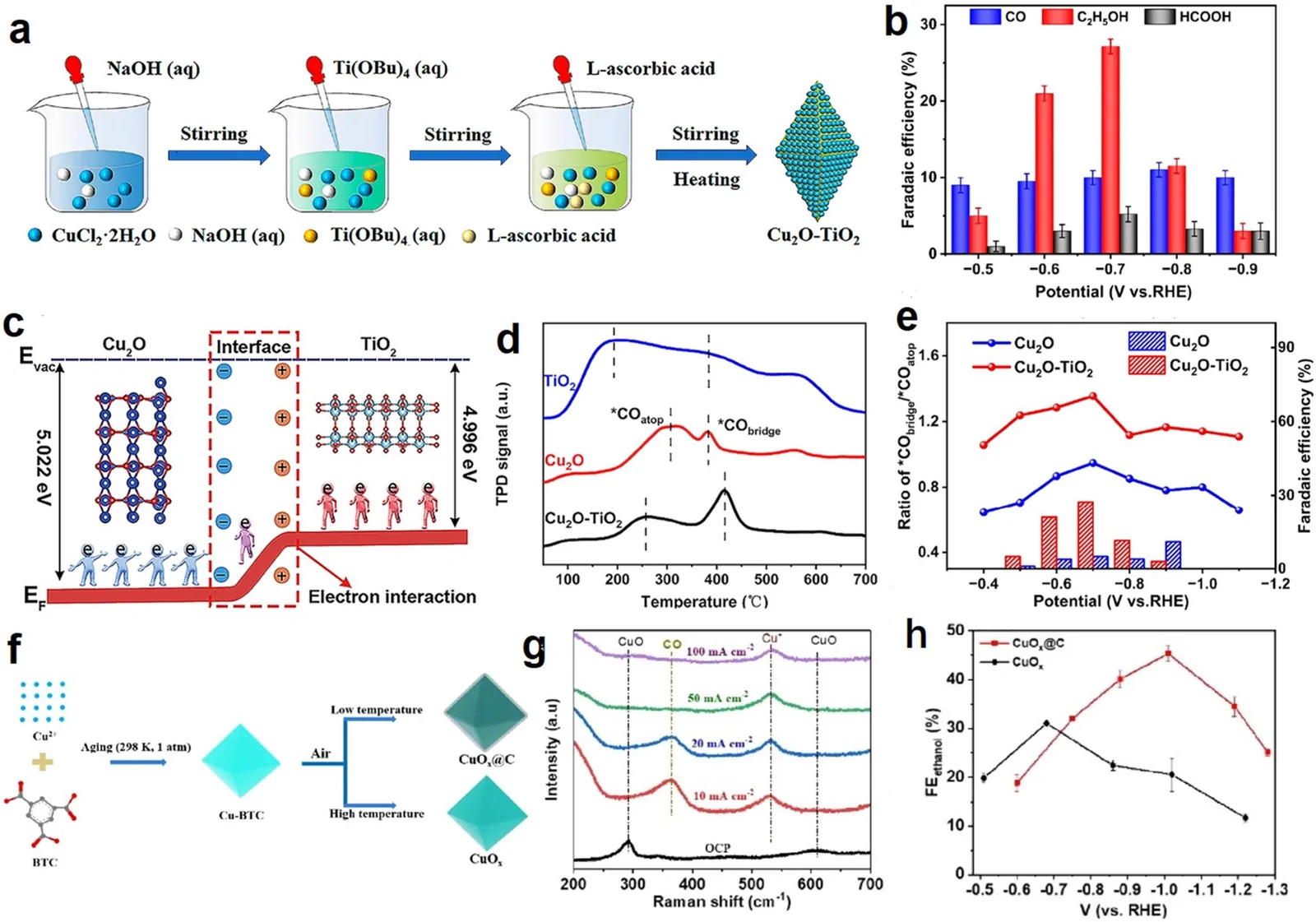
**a** Schematic illustration of Cu_2_O–TiO_2_ synthesis. **b** CO, C_2_H_5_OH and HCOOH FEs of Cu_2_O-TiO_2_ at varying applied potentials during an hour of electrolysis in 0.5 M KHCO_3_ electrolyte. **c** Schematic diagram of the electron interaction between Cu_2_O and TiO_2_. **d** CO-TPD spectra for Cu_2_O, Cu_2_O-TiO_2_ and TiO_2_. **e** Potential-dependent ratios of CO_bridge_ to CO_atop_ and the corresponding FEs of ethanol for Cu_2_O and Cu_2_O-TiO_2_ [129]. Copyright 2023, American Chemical Society. **f** Illustration of CuO_x_@C and CuO_x_ electrocatalysts preparation. **g** Operando Raman spectra during eCO_2_RR under different current densities of CuO_x_@C. **h** Potential-dependent Faradaic efficiencies of ethanol over CuO_x_@C and CuO_x_ electrocatalysts [133]. Copyright 2022, Wiley–VCH

In general, when metal Cu is placed on some substrate materials, the interface between Cu and the substrate will produce some Cu^+^ and stabilize it due to its strong interaction, as a way to increase the CO coverage on the electrocatalyst surface and strengthen the kinetics of C–C coupling [126–128]. For example, Yan’s team designed Cu_2_O–TiO_2_ heterostructured electrocatalysts to modulate the CO adsorption configuration to improve the ethanol selectivity (Fig. 11a) [129]. At a potential of − 0.7 V vs. RHE, Cu_2_O–TiO_2_ exhibited a Faradaic efficiency of 27.13% for C_2_H_5_OH (Fig. 11b). A schematic of the electronic interactions between Cu_2_O and TiO_2_ showed that there was a strong electronic interaction between Cu_2_O and TiO_2_, which led to an increase in the valence state of Ti, thus enhancing the pro-oxidant nature of TiO_2_ (Fig. 11c). The effect of electronic interactions between Cu_2_O and TiO_2_ on the CO adsorption configuration in Cu_2_O–TiO_2_ was then investigated using CO–TPD and in situ Raman spectroscopy, and Fig. 11d shows that the CO_bridge_ desorption peaks appeared in Cu_2_O-TiO_2_ at higher temperatures, which suggests that Cu_2_O–TiO_2_ has a strong bonding strength with CO_bridge_. This behavior can be attributed to the strong oxygenophilicity of TiO_2_. Furthermore, these bound CO configurations were also quantified, as shown in Fig. 11e, where the CO_bridge_/CO_top_ ratio of Cu_2_O–TiO_2_ was significantly higher than that of Cu_2_O in the detected potential range and the electrocatalysts with different CO_bridge_ and CO_top_ ratios exhibited a volcano type relationship for the FEs of ethanol, which suggests that increasing the adsorption level of CO_bridge_ could promote the formation of ethanol. These findings suggest that TiO_2_ doping with Cu_2_O significantly enhanced CO_bridge_ adsorption and thus promoted ethanol generation. This study also provides original insights into the design of highly ethanol-selective electrocatalysts by engineering CO adsorption structures.

In addition to conventional substrate materials such as TiO_2_, carbon shells can also efficiently stabilize numerous Cu^+^ species, thus facilitating the C–C coupling step by improving *CO adsorption. In contrast, metal–organic frameworks (MOFs) materials possess the benefits of rich structure, ultralarge specific surface area, and porous structure, as well as diverse chemical functions, which can be utilized as electrocatalysts by themselves or as precursors for further create electrocatalysts with superior performance [130–132]. Therefore, Wang et al. [133] developed a simple carbon coating strategy to prepare CuO_x_@C electrocatalysts with carbon coatings by one-pot pyrolysis of copper-based MOF (Fig. 11f). Then eCO_2_RR was carried out in a flow cell, and it was found that the FE of C_2_H_5_OH on the electrocatalyst showed a volcano type relationship at different potentials, with the highest FE of C_2_H_5_OH reaching 46% at a potential of -1.0 V vs. RHE (Fig. 11h). Then, quasi-in situ Raman is used to further define the role of the carbon coatings. As shown in Fig. 11g, a peak of 532.3 cm^−1^, belonging to Cu^+^, appears at CuO_x_@C, and Cu^+^ can be stabilized under a broad current density window, while Cu^+^ does not exist in CuO_x_. The results indicate that the carbon coating stabilizes the Cu^+^ species in CuO_x_@C during eCO_2_RR, while the Cu^+^ species favors the formation of CO at low current densities and promotes C–C coupling at high current densities. Furthermore, DFT calculations reveal that carbon coating can promote the adsorption of intermediate CO on the active site to facilitate C–C coupling, and it can also modulate the hydrogenation pathway of intermediate *HOCCH so that the reaction proceeds toward the production of C_2_H_5_OH.

In short, the modulation of Cu oxidation state can effectively increase the Cu^+^ content and stability of the electrocatalysts, thus showing better ethanol performance and catalytic stability.

#### Bimetallic Electrocatalysts

Bimetallic electrocatalysts are novel catalysts created by combining two metal elements into one catalyst [134]. By doping other metals, not only the geometry and electronic structure of the electrocatalyst material can be changed, but also new reactive active sites can be provided, thus optimizing the binding strength between the reaction intermediates and the active sites [135, 136]. The bimetal’s synergistic effect in the eCO_2_RR process can alter the adsorption energy of the intermediate products, and alloying Cu with another metal can stabilize Cu nanoparticles to achieve high ethanol yield [137]. Not only that, combining bimetallic electrocatalysts with crystal surface engineering and defect engineering can also assist modify the active site’s electronic structure, optimize the energy barrier of the C–C coupling process, and enhance the selectivity of C_2+_ products such as ethanol [138]. As mentioned earlier, in the eCO_2_RR process, the FE of ethanol is lower than that of ethylene in most cases because ethanol and ethylene share an identical key intermediate, *CO–*COH, and ethanol has a higher energy barrier. If one of the C–O bonds in the *CO–*COH intermediates must unchanged if ethanol is to be produced, then one of the well-tested strategies is to coordinate the metal ion with one of the two C_1_ intermediates via the oxygen atom, which prevents oxygen atoms from hydrogenating prematurely and allows the C–O bond to remain, helping the reaction to move toward the ethanol pathway [45]. In addition, asymmetric C–C coupling between the other *CO intermediates and *OCH_2_ intermediate can generate the critical intermediate *CO–*OCH_2_, which is more favorable for ethanol production than ethylene [121, 139].

**Fig. 12:**
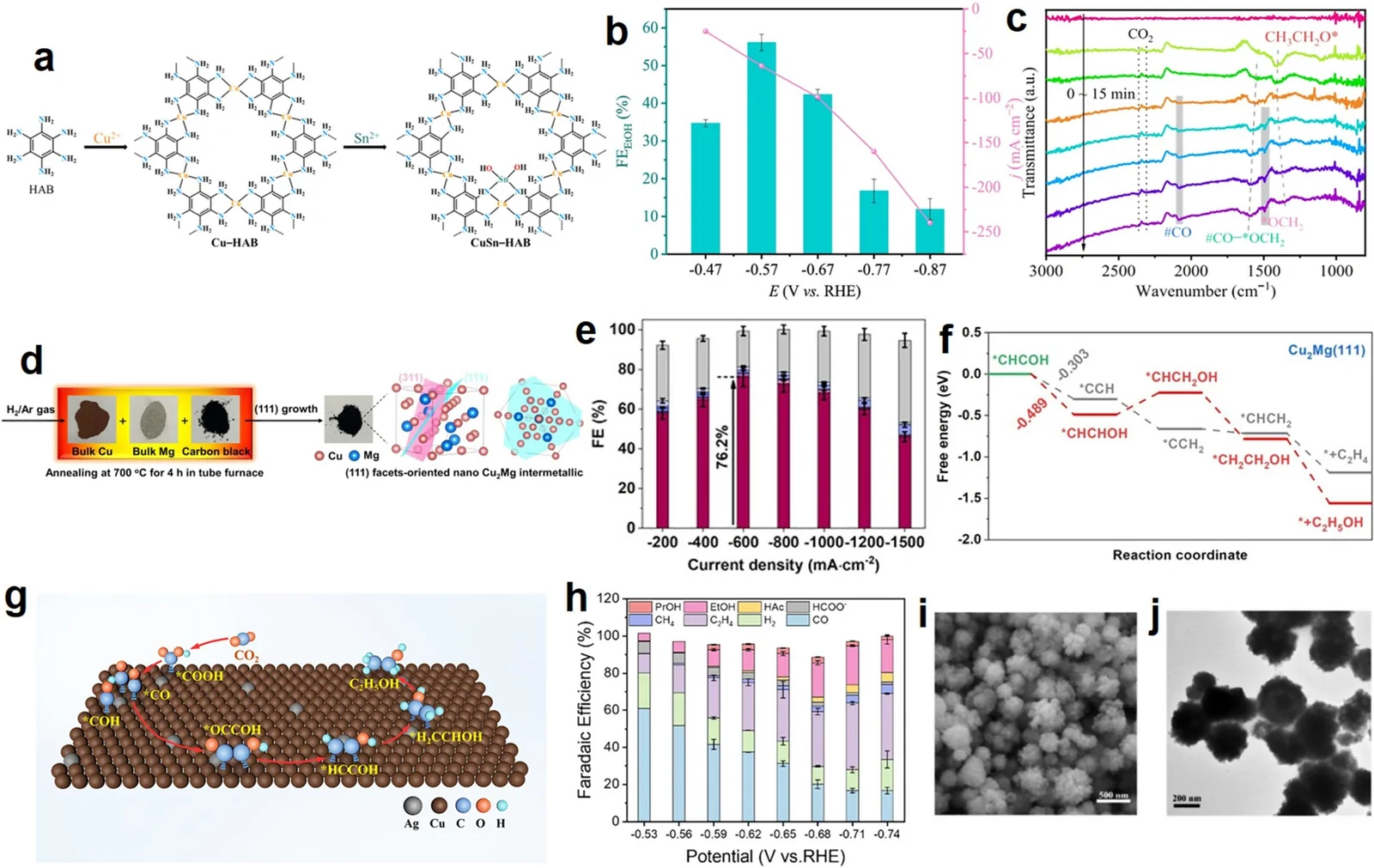
**a** Illustration of the synthetic process for preparing CuSn–HAB. **b** FE_EtOH_ values at different potentials by CuSn–HAB as electrocatalyst. **c** Time-dependent operando ATR-FTIR spectra for eCO_2_RR on CuSn–HAB at − 0.57 V vs RHE in 1 M KOH electrolyte [66]. Copyright 2023, American Chemical Society. **d** Schematic of synthesizing (111) facet-oriented nano-Cu_2_Mg intermetallic compound using Cu, Mg, and carbon powder. **e** Faradaic efficiencies (FE) of CO_2_ reduction and H_2_ products for the Cu_2_Mg(111) electrocatalyst. **f** Energy diagrams of hydrogenation of *CHCOH intermediate to form ethanol and ethylene on Cu_2_Mg(111) [145]. Copyright 2024, Wiley–VCH. **g** Proposed possible reaction mechanism of CO_2_ electroreduction to ethanol on a Ag-doped Cu surface. **h** Faradaic efficiency of CO_2_ reduction products at different applied potentials on CuAg-0.75%. Morphological characterization of the as-prepared CuAg-0.75% sample: **i** SEM and **j** TEM images [159]. Copyright 2022, American Chemical Society

Based on this idea, Chen and his colleagues successfully synthesized a MOF containing unique heterogeneous bimetallic sites of CuN_4_ and SnN_2_O_2_ by PSM treatment (CuSn-HAB) (Fig. 12a) [66]. After CO_2_ reduction performance testing, CuSn-HAB achieved a FE of 56(2)% for the conversion of eCO_2_RR-to-ethanol at a high current density of 68 mA cm^−2^ and an ultrahigh-energy conversion efficiency of 35.5% (Fig. 12b), and could be stabilized for more than 35 h, which represents the excellent performance of eCO_2_RR-to-ethanol to date. The Time-dependent operando ATR-FTIR spectrum of Fig. 12c shows a wide range of different intermediates that are essential for promoting the asymmetric C–C coupling [140]. Meanwhile, the #CO–*OCH_2_ intermediate at 1583 cm^−1^ and the CH_3_CH_2_O* intermediate at 1398 cm^−1^ are the key intermediates in charge of the production of ethanol [141]. The results demonstrate that the SnN_2_O_2_ site in the heterometallic asymmetric doublet has high affinity for O atoms, which is helpful for the production of *OCH_2_ key intermediates and stabilization of their C–O bonds. Thus, the asymmetric C–C coupling between *CO and *OCH_2_ intermediates occurring at this heterometallic asymmetric double site is thermodynamically favorable compared to conventional homometallic or symmetric double sites, thus exhibiting higher selectivity for ethanol rather than ethylene. This work also lays the foundation for the subsequent design of electrocatalysts with multiple reactive active sites to convert CO_2_ into high-value multicarbon products with specific targets [66].

The secondary metals reported to alloy with copper are mainly focused on eCO_2_RR reactive metal elements such as Au, Ag, Zn, Sn, and Pd [142–144]. In contrast, much less attention has been paid to alkali or alkaline earth metal elements due to their inherent chemical activity that reacts easily with water or air [145]. Among them, magnesium (Mg) is relatively inert at room temperature. The multivalent nature and smaller cation radius of Mg proved to have stronger interactions with *CO_2_^−^ intermediates and to dissociate more readily by hydrolysis than bases or other alkaline earth metals [146, 147]. The monoatomic Mg-coordinated carbon electrocatalysts showed near-optimal binding strength to oxygen-containing substances through the rise of the p-band center position [148]. On this basis, Zheng et al. [145] suggested that controlling the Mg doping and coordination environment might help to improve the *CO coverage of the Cu catalytic center and stabilize the oxygenated ethanol intermediates, thus greatly improving the CO_2_-to-ethanol selectivity. They developed a (111)-face-exposed Cu_2_Mg electrocatalyst with densely ordered Cu_3_-Mg sites to induce electron transfer from Mg to Cu (Fig. 12d). Electrocatalytic eCO_2_RR tests were carried out in a flow cell with 1 M KOH aqueous solution at atmospheric pressure. The Cu_2_Mg(111) electrocatalyst demonstrated a high ethanol Faradaic efficiency of 76.2% ± 4.8% at 600 mA cm^−2^ (Fig. 12e), with a peak ethanol local current density (j_C2H5OH_) of 720 ± 34 mA cm^−2^, which is almost as high as that of the conventional four times that of the Cu_2_Mg(311) electrocatalyst, which is comparable to the best value reported for ethanol production by eCO_2_RR. This work is the first to apply DFT calculations to the study of *COCO coupling, which is considered to be the critical step that determines the rate for C_2+_ production. In contrast to Cu_2_Mg(311), the Cu_2_Mg(111) model has a lower energy barrier and reaction energy, indicating faster kinetics and more favorable thermodynamics. And it is able to stabilize the *CHCHOH intermediate, thus facilitating the ethanol formation pathway (Fig. 12f).

Since CO is a essential intermediate product in the reduction of CO_2_ to C_2+_, effective cascading of the two processes CO_2_ → CO and CO → C_2+_ has become a novel strategy that has attracted much attention recently [149–151]. Therefore, combining CO-producing electrocatalysts with Cu electrocatalysts that can reduce CO deeply has become an effective means to improve C_2+_ selectivity. Compared with single-site catalyzed CO_2_ reduction to C_2+_ products, two-site tandem catalysis is more efficient [152–154]. During the eCO_2_RR process, the overflow of CO produced by the second metal element to the Cu surface leads to higher *CO coverage, which reduces the energy barrier for *CO coupling, and thus makes it easier to form C_2+_ products such as ethanol [155].

Among them, CuAg bimetallic compounds show promise as electrocatalysts for the eCO_2_RR-to-ethanol due to their adjustable electronic structure and exposure of the reactive active site [156–158]. The study of Bo-Lin Lin’s team reported Ag-modified Cu_2_O materials to improve the catalytic performance of CO_2_ electroreduction to ethanol [159]. Images from SEM and TEM of CuAg-0.75%, shown in Fig. 12i, j, show that the compound has a roughly spherical shape with a size of 330 nm. In addition, their synthesized CuAg-0.75% electrocatalyst achieved an ethanol FE of 21.0% at − 0.71 V vs. RHE and a maximum ethanol partial current density of 214.4 mA cm^−2^ at − 0.74 V vs. RHE, which is about twice as much as that of its counterpart without Ag modification (Fig. 12h). DFT calculations showed that the Ag-modified Cu surface exhibited a stronger CO adsorption strength is stronger than that on the Ag surface and, therefore, can promote the overflow of *CO from the Ag sites to the Cu sites, resulting in a higher *CO coverage than that on the pure Cu surface, which will help with the C–C coupling process. The uniformly distributed Ag atoms provide a large number of Ag sites and short CO transfer distance for CO formation. And the Ag-doped samples formed the essential intermediates of the ethanol pathway with lower free energies, which improved the selectivity of ethanol. Both the enhanced CO overflow process and the optimization of the free energy on the Ag-modified samples led to better catalytic performance for ethanol (Fig. 12g).

In summary, the selectivity and activity of the electrocatalysts toward ethanol have been significantly improved by the bimetallic tandem strategy.

**Fig. 13:**
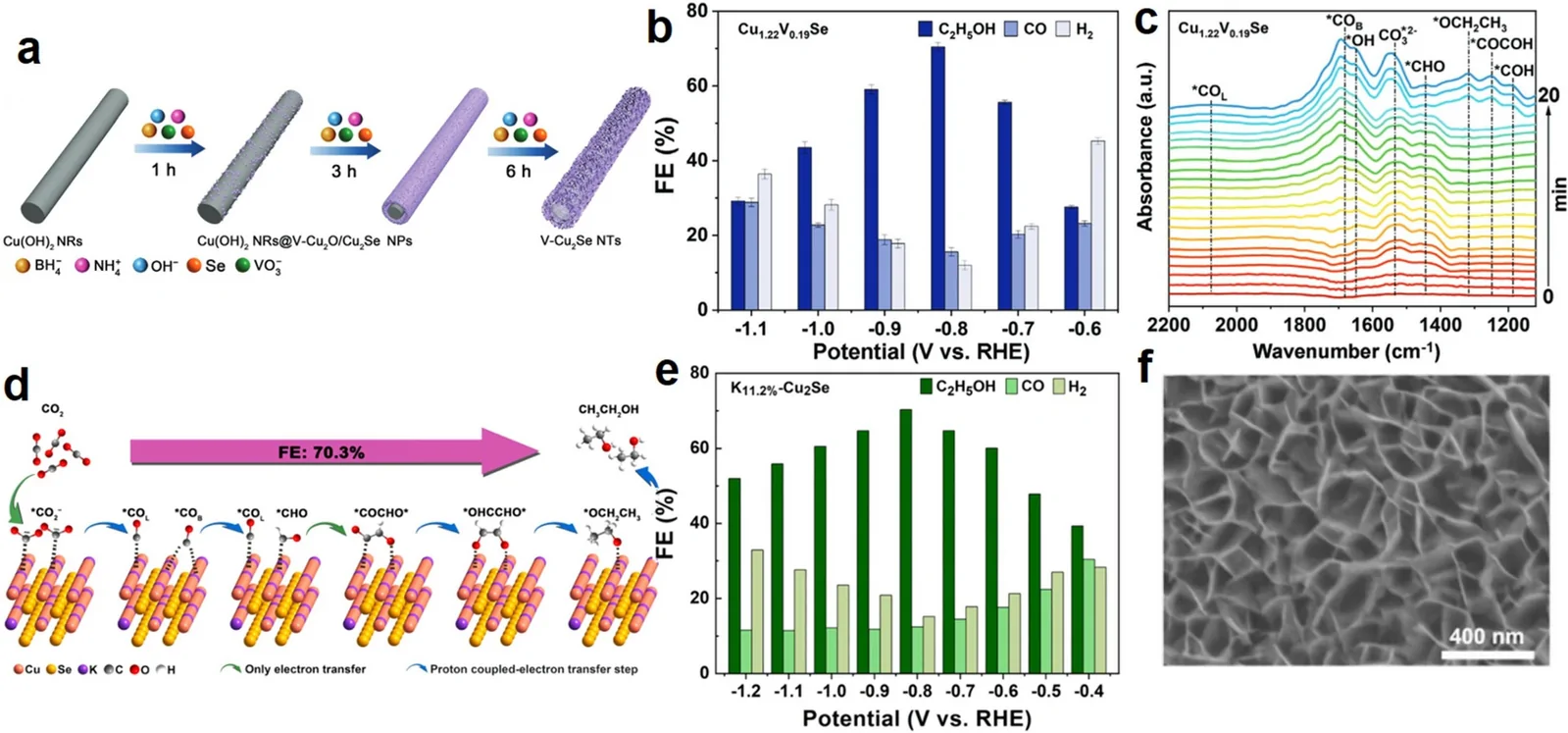
**a** Schematic illustration for the growth process of V-doped Cu_2_Se nanotubes. **b** FEs of various products by Cu_1.22_V_0.19_Se. **c** Time-dependent in situ DRIFTS spectra for eCO_2_RR on Cu_1.22_V_0.19_Se [162]. Copyright 2022, Wiley–VCH. **d** Schematic illustration for the catalytic mechanism for ethanol production in eCO_2_RR on K-doped Cu_2_Se. **e** FEs of ethanol, CO, and H_2_ for K_11.2%_-Cu_2_Se at different potentials. **f** SEM image of K_11.2%_-Cu_2_Se [61]. Copyright 2022, Wiley–VCH

Beyond modulating intermediate adsorption, the presence of Cu⁺ on the electrocatalyst surface also improves the efficiency and selectivity of eCO_2_RR [160], but in the actual electrolytic environment, Cu^+^ is easily reduced, resulting in a Cu^0^-dominated physical phase of the electrocatalyst [161]. Based on this, Xiong et al. [162] designed V-doped Cu_2_Se hierarchical nanotubes for the synthesis of a single liquid ethanol product by eCO_2_RR in a flow cell (Fig. 13a). Doping the Cu_2_Se lattice with V^4+^ ions diversified the active sites and protected the Cu^+^ species from reduction to Cu^0^ in the eCO_2_RR process. After CO_2_ reduction performance testing, as shown in Fig. 13b, the optimal Cu_1.22_V_0.19_Se nanotubes generated C_2_H_5_OH with a selectivity of 68.3% by eCO_2_RR. As demonstrated by the in situ DRIFTS spectroscopy in Fig. 13c, V^4+^ doping diversified the active sites, altered the local charge distribution of Cu_2_Se, and protected the Cu^+^ species during the eCO_2_RR process. The special reactive active sites promoted the formation of bridge *CO_B_ and facilitates the hydrogenation process to generate *COH intermediates, and *COH is subsequently coupled with *CO_L_ to ultimately produce ethanol. This work provides significant insights to steer the reaction path toward the design of electrocatalytic materials for high-performance ethanol production and opens the way for flow cell eCO_2_RR to a single C_2+_ liquid fuel.

Following this line of thought, their research group then successfully prepared a K-doped Cu_2_Se nanosheet electrocatalyst supported on copper foam to achieve highly selective generation of ethanol by modulating the interaction between Cu sites and reaction intermediates in eCO_2_RR (Fig. 13f) [61]. Combined with the correlation characterization and DFT calculations, it was found that electron transfer from K to Se stabilizes the Cu(I) intermediate species, which promotes the adsorption of linear *CO_L_ and bridged *CO_B_ intermediates, which in turn synergistically promotes the C–C coupling in the eCO_2_RR process (Fig. 13d). Consequently, the optimized 11.2% K-Cu_2_Se nanosheet electrocatalyst could achieve CO_2_ reduction to ethanol with high selectivity in the overpotential interval from − 0.6 to − 1.2 V. The optimized ratio of 11.2% K-Cu_2_Se nanosheet electrocatalyst could be used to reduce CO_2_-to-ethanol with high selectivity. In addition, the 11.2% K-Cu_2_Se nanosheet electrocatalyst achieved a local current density of 35.8 mA cm^−2^ at an overpotential of − 0.8 V vs. RHE, a Faradaic efficiency of 70.3% for of eCO_2_RR-to-ethanol, and an excellent stability of 130 h in 0.1 M KHCO_3_ (Fig. 13e). At the same time, the FE of ethanol reached more than 50% over a wide potential interval from − 0.6 to − 1.2 V, indicating that the electrocatalyst has significant potential for practical applications.

All in all, in addition to the direct series of bimetals, the evolution of key reaction intermediates can be controlled by doping alkali metal cations in Cu compounds, so as to enhance the catalytic effect of eCO_2_RR on ethanol.

### Non-Copper-Based Electrocatalysts

To date, most of the electrocatalysts found to be capable of generating C_2+_ products from eCO_2_RR at appreciable reaction rates are copper-based materials, while it can be found, based on much of the work presented in the previous section, that this is owing to the strong adsorption of *CO intermediates on the copper surface facilitating the formation of the C–C bond through CO dimerization or COCHO coupling. However, within the recent past, successive groups have reported that non-Cu-based electrocatalysts for CO_2_ reduction can also produce ethanol, and they have done a large number of experiments and demonstrated, by means of different characterizations, that non-copper-based electrocatalysts can yield ethanol from a different coupling pathway than that of copper-based electrocatalysts.

**Fig. 14:**
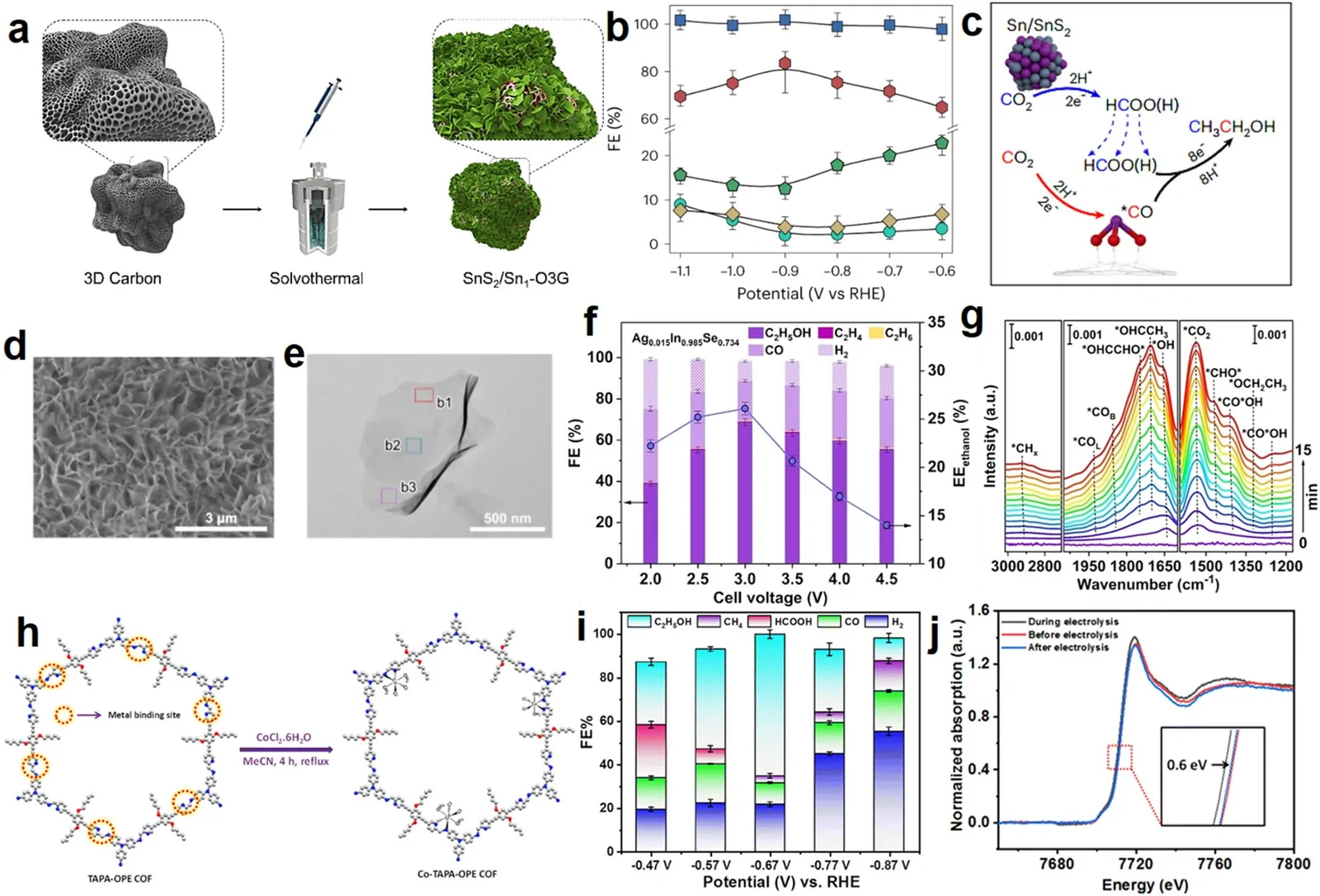
**a** A schematic illustration showing the procedure to prepare SnS_2_@Sn_1_-O3G. **b** FE values with error bars indicated (the FE was measured in a 2-h interval at each potential, and the y axis is broken from 25% to 58%, to show the FF of all products clearly). **c** A schematic illustration showing the cascade reaction during CO_2_ reduction to ethanol over SnS_2_/Sn_1_-O3G (gray: S, red: O, yellow: H and purple: Sn) [67]. Copyright 2023, Nature Publishing Group. **d** SEM image and **e** TEM image of Ag_0.015_In_0.985_Se_0.734_. **f** FEs of the products and EEs of ethanol on Ag_0.015_In_0.985_Se_0.734_ at different cell voltages. **g** Time-dependent in situ DRIFTS spectra of Ag_0.015_In_0.985_Se_0.734_ at − 0.6 V [171]. Copyright 2023, Wiley–VCH. **h** Synthesis of Co^2+^ metalated TAPA-OPE COF. **i** Product distribution plot during eCO_2_RR at different potentials. **j** Co K-edge XANES spectra for Co-TAPA-OPE COF before, during, and after electrolysis. The inset shows the enlarged Co K-edge XANES spectra [177]. Copyright 2024, The Royal Society of Chemistry

Recently, Huang and coworkers developed a tandem electrocatalyst consisting of SnS_2_ nanosheets and a single Sn atom coordinated with three oxygen atoms on a 3D carbon [67]. As shown in Fig. 14a, they first prepared 3D carbon foam as a carrier by depositing C_2_H_4_ onto the HY zeolite skeleton, which can increase the surface area of the carrier and obtain excellent mass transfer effect. Then SnS_2_/Sn_1_-O3G was prepared by solvothermal reaction using SnBr_2_ as Sn source and thiourea as *S* source. The electrocatalyst produces ethanol repeatedly with a FE of up to 82.5% and a geometric current density of 17.8 mA cm^−2^ at − 0.9 V Vs. RHE (Fig. 14b). Meanwhile, the Faradaic efficiency of the electrocatalyst for ethanol production remained above 70% throughout the potential range of − 0.6 to − 1.1 V Vs. RHE, and the electrocatalyst continued to exhibit excellent ethanol performance after continuous reaction at -0.9 V Vs. RHE for up to 100 h. Notably, no other C_2_ products were detected at all potentials applied in the eCO_2_RR. Besides, combined with isotope labeling experiments and density functional theory studies, it was proved that the dual active center including Sn and O atoms can adsorb *CHO and *CO(OH) intermediates, respectively, thus promoting the formation of C–C bonds through the formyl-bicarbonate coupling pathway (Fig. 14c). The present work overcomes the limitations of Cu-based electrocatalysts and develops a new strategy for obtaining highly selective ethanol by modulating the CO_2_ reduction pathway, which lays the foundation for subsequent studies on the production of multicarbon products over non-Cu-based electrocatalysts.

In addition, the electroreduction of CO_2_-to-ethanol at a high current densities is an attractive method of carbon dioxide utilization [64, 163, 164]. And the membrane electrode assembly (MEA) electrolyzer can greatly shorten the distance between the electrodes, eliminate the cathodic electrolyte, and improve the energy efficiency to realize eCO_2_RR applications at high current density and low-energy consumption [165–168]. However, the commonly used Cu-based electrocatalysts in MEA electrolyzer are prone to in situ electroreduction of the active species (Cu^δ+^) despite their ethanol-generating ability, which makes it difficult to maintain high currents during the eCO_2_RR process [169, 170]. Therefore, Xiong et al. [171] designed and synthesized a non-copper-based Ag^+^ doped InSe nanosheets with Se vacancies by using InSe, which has the advantages of good electrical conductivity, large surface area, excellent mechanical properties, and stable physicochemical properties, to meet this challenge through heteroatom doping, adjusting the electronic structure of the original active sites and introducing new active sites to optimize the adsorption and desorption behaviors of the intermediates.

The Ag_0.015_In_0.985_Se_0.734_ synthesized by them demonstrates the morphology of flexible nanosheets with a transverse size of around 500 nm by SEM and TEM images, and has a polycrystalline structure with obvious grain boundaries (Fig. 14d, e). Then, by coupling the cathodic eCO_2_RR with the anodic oxygen precipitation reaction (OER) in the MEA electrolyzer, the optimized Ag_0.015_In_0.985_Se_0.734_ nanosheets achieved an C_2_H_5_OH Faradaic efficiency of 68.7% and a partial current density of 186.6 mA cm^−2^ at 3.0 V, with a full-cell energy efficiency of 26.1%, and a 22-h long-term stability (Fig. 14f). As shown in Fig. 10g, they also demonstrated by time-dependent in situ DRIFTS spectra and DFT calculations that the synergistic effect of Ag and In double sites largely promoted the generation of *CO*OH intermediates, in which C atoms adsorbed on Ag sites and O atoms bonded on In sites formed a special adsorption conformation, which effectively stabilized the *CO*OH intermediates, and contributed to their further conversion to *CO_L_ and *CO_B_ intermediates with enhanced adsorption strength. This can promote the hydrogenation of *CO_L_ intermediates to *CHO* intermediates and the coupling with *CO_B_ intermediates on Ag–Ag bridge sites to generate *COCHO* intermediates, and ultimately *COCHO* intermediates to generate ethanol from the thermodynamically most favorable pathway. At the same time, Ag^+^ doping can better protect In^2+^ during the eCO_2_RR process (Fig. 14g). This work not only designed an efficient non-copper-based electrocatalyst for the preparation of ethanol by electroreduction of CO_2_, but also provided insights into the catalytic active sites, which guided the subsequent synthesize of more highly selective and energy-efficient electrocatalysts for the preparation of ethanol by eCO_2_RR at high current density.

In a distinct material strategy, covalent organic frameworks (COFs), a class of crystalline organic porous polymers, are made up of organic motifs joined by covalent bonds. COFs have a high specific surface area, low density, and flexible structural design, and they can be used in a variety of processes like substance adsorption, multiphase catalysis, sensing, and photovoltaics [172–176]. Based on its excellent electrocatalytic properties, Prof. Tapas Kumar Maji et al. [177] successfully prepared a redox-active covalent organic framework (COF), TAPA-OPE, through the dialdehyde condensation of triamino-triphenylamine (TAPA) and oligo(p-phenylene acetylene) (OPE). Then, in order to further explore the metal chelating ability of TAPA-OPE toward electrocatalytically active metal ions, Co^2+^, which has superior electrochemical activity and abundant as well as low price, was selected for COF-based CO_2_ electroreduction. Accordingly, they used TAPA-OPE for covalent grafting of Co^2+^ to synthesize Co-TAPA-OPE (Fig. 14h). It is noteworthy that this material is also based on a non-copper-based electrocatalyst. Afterward, they evaluated the eCO_2_RR performance of the material. Specifically, the performance was tested by adding 0.2 M KHCO_3_ electrolyte solution to the H-type cell, and it was found that the Co-TPA OPE could selectively reduce CO_2_ to C_2_H_5_OH at -0.67 V vs. RHE, with a Faradaic efficiency of up to 66.8% (Fig. 14i). Upon in situ XAS studies as shown in Fig. 14j, it was shown that individual Co active sites instantaneously change their oxidation states and coordination environments during electrochemical reduction. Moreover, they performed in situ FTIR studies to observe various intermediates during the eCO_2_RR, which helped to elucidate the reaction mechanism by DFT calculations. This study could pave the way for the development of inexpensive and efficient COF-based electrode materials and be used to convert CO_2_ into high-value ethanol products, which ultimately hold the promise of carbon neutrality.

**Fig. 15:**
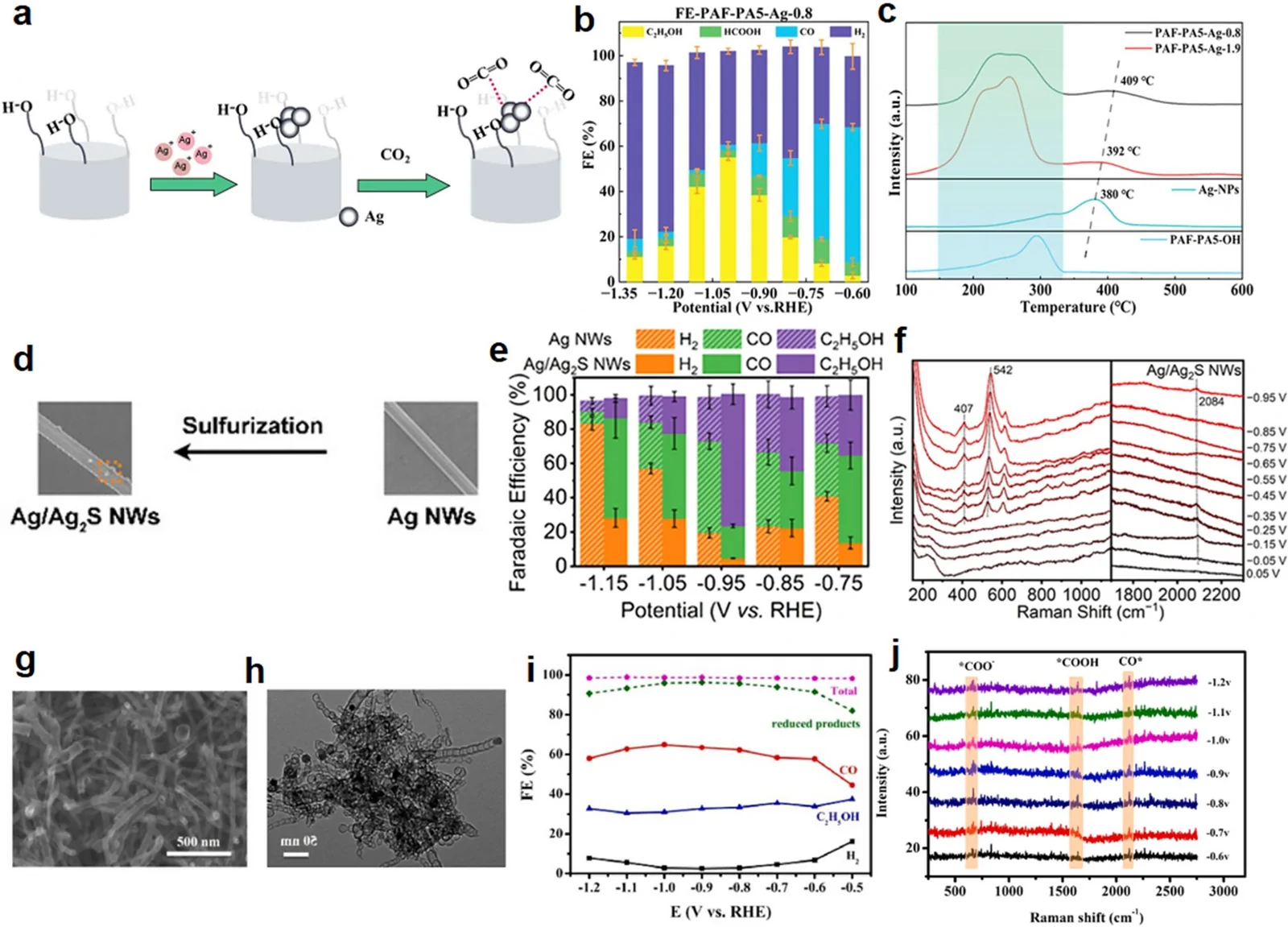
**a** Cartoon illustration of the preparation of the electrocatalysts PAF-PA5-Ag-0.8 and PAF-PA5-Ag-1.9 and their adsorption of CO_2_. **b** Product distributions on PAF-PA5-Ag-0.8. **c** CO-TPD spectra of PAF-PA5-Ag-0.8, PAF-PA5-Ag-1.9, AgNPs, and PAF-PA5. The peaks in the shaded range were derived from decomposition of PAF-PA5-OH at elevated temperatures [181]. Copyright 2023, Wiley–VCH. **d** Schematic diagram of Ag NWs sulfidation into Ag/Ag_2_S NWs. **e** FE of different products during CO_2_ electroreduction on Ag NWs and Ag/Ag_2_S NWs. **f** SERS spectra of CO_2_ electroreduction on Ag/Ag_2_S NWs at different potentials in CO_2_-saturated 0.5 M KHCO_3_ [182]. Copyright 2025, Wiley–VCH. **g** SEM and **h** TEM images of Ni@NCNT-700. **i** FE plot for Ni@NCNT-700. **j** In situ Raman spectra at the potential range from − 0.6 to − 1.2 V of Ni@NCNT-700 [184]. Copyright 2022, Elsevier B.V

In eCO_2_RR, CO is usually the dominant product over Ag-based electrocatalysts, which may result from the weak adsorption of *CO [178–180]. In this study, Yang and his colleagues prepared a hydroxyl columnar aromatic extended porous polymer-constrained Ag electrocatalyst (PAF-PA5-Ag-0.8) for electrocatalytic eCO_2_RR (Fig. 15a) [181]. As shown in Fig. 15b, this electrocatalyst can selectively produce C_2_H_5_OH at a current density of 11 mA cm^−2^ with a FE of up to 55%. It was shown that the hydroxyl columnar aromatic-restricted Ag cluster is the active site for C_2_H_5_OH formation. In addition, programmed warming desorption experiments showed that the CO desorption intensity of the main sample at 409 °C was increased compared to both PAF-PA5-Ag-1.9 and commercial AgNPs, which proved that the CO adsorption intensity of the PAF-PA5-Ag-0.8 sample was the highest among the three electrocatalysts. Therefore, this favors the C–C coupling process and thus the highly selective formation of C_2_H_5_OH (Fig. 15c). DFT studies showed that the restricted Ag clusters in the main sample could exhibit high ethanol selectivity for eCO_2_RR by promoting the formation of *COOH, stabilizing the *CO intermediates, and inhibiting the HER. The research offers a novel design approach to modulate the adsorption strength of *CO on non-Cu electrocatalysts, therefore converting CO_2_ into "green" multicarbon products.

Li’s research group employed a controlled sulfidation strategy to construct atomically designed Ag/Ag_2_S nanowires (NWs) for eCO_2_RR (Fig. 15d) [182]. As shown in Fig. 15e, at a potential of -0.95 V vs. RHE, sulfidation increased the FE of C_2_H_5_OH from 25% to 75% and achieved exceptional stability exceeding 14 h, outperforming most reported Ag-based systems. In situ electrochemical surface-enhanced Raman spectroscopy (EC-SERS) revealed key signals at 2084 cm^−1^ for *CO and 542 cm^−1^ for *CH_2_CHO on the Ag/Ag_2_S surface (Fig. 15f). In contrast, Ag NWs only exhibit a formic acid signal at 1761 cm^−1^. Combined with DFT calculations, it was demonstrated that the Ag/Ag_2_S heterointerface synergistically regulates the interfacial water network and stabilizes the critical *CO intermediate, thereby accelerating CO_2_ activation, proton-coupled electron transfer, and asymmetric C–C coupling. Additionally, the sulfur-induced dual effect—optimized hydrogen bonding interactions and enriched K⁺ confinement—was identified as the key driver regulating the local microenvironment to enhance ethanol selectivity. This work not only demonstrates rational atomic interface design targeting C_2_ products but also deciphers the dynamic interactions between electrocatalyst electronic structure and interface species, providing a molecular-level roadmap for advanced CO_2_ conversion systems.

In addition to the previously reported work, the d-band state is decreased when transition metal clusters are encapsulated in carbon nanotubes (CNTs) [183]. Based on this, Jun Wang’s group reported the electrosynthesis of ethanol over non-Cu electrocatalysts via eCO_2_RR in the presence of Ni@NCNT electrocatalysts, which bonded and confined Ni nanoparticles with N-doped carbon nanotubes (Ni@NCNT) [184]. Using dicyandiamide as a source of carbon and nitrogen, they used the chemical vapor decomposition (CVD) approach to synthesized Ni@NCNT electrocatalysts on nickel foam in an argon atmosphere. Ni residues were completely removed from the obtained samples by carefully washing them in a 0.5 M HCl solution following calcination at various temperatures from 600 to 900 °C. As shown in Fig. 15g, the outer surface of Ni@NCNT-700 exhibited a worm-like tubular morphology in the absence of Ni nanoparticles (Ni NPs), clarificating that the CNTs were successfully prepared by CVD and the Ni was completely removed. According to the TEM image of Fig. 15h, individual Ni nanoparticles could be seen at the head of each N-doped CNTs on the entangled Ni@NCNT-700 network. The electrocatalytic performance of eCO_2_RR on Ni@NCNTs was subsequently assessed in a H-cell. As shown in Fig. 15i, the three eCO_2_RR products on the main sample are CO, C_2_H_5_OH, and H_2_. It is noteworthy that the maximum FE for C_2_H_5_OH reaches 38.5% at − 0.5 V vs. RHE and stays above 30% over a wide potential interval from − 0.5 to − 1.2 V. Then, in situ Raman spectroscopy was used to observe various intermediates in the eCO_2_RR process. As shown in Fig. 15j, the characteristic peaks at about 720, 1650, and 2050 cm^−1^ can be attributed to *COO^−^, *COOH, and *CO intermediates, which are particularly important for the conversion of CO_2_ to C_2_H_5_OH. Finally, according to the DFT calculation, it is proved that the constraints and synergies of N kinds on NCNTs and Ni NPs can lead to a lowering of the energy barrier of C–C coupling, and at the same time, it also hinders the desorption of CO and inhibits HER, so that CO_2_ can be efficiently reduced to C_2_H_5_OH.

To summarize, the electrocatalysts used for CO_2_ reduction of ethanol are still dominated by copper-based electrocatalysts, and the research work reported on CO_2_ production of ethanol on non-copper-based electrocatalysts is very limited, but their work proves that the production of C_2+_ products on non-copper-based electrocatalysts is feasible, which lays a solid foundation for our subsequent research.

## Conclusions and Perspectives

In summary, the process of electrocatalytic CO_2_ reduction (eCO_2_RR) to ethanol presents a viable approach to transforming waste CO_2_ into valuable chemicals or fuels, which has a great potential for achieving carbon neutrality goals. In this article, based on the existing applied research results on the production of multicarbon products via eCO_2_RR, we review the recent research advances in the eCO_2_RR-to-ethanol on copper-based electrocatalysts and non-copper-based electrocatalysts. Section 2 analyzes economic feasibility of ethanol product, demonstrating that it is an economically and technologically viable pathway for achieving ethanol product synthesis via CO_2_ electroreduction. Section 3 summarizes the catalytic mechanism of ethanol production by eCO_2_RR, which is hoped to provide readers with a more intuitive understanding in terms of the mechanism. Section 4 discusses in detail the copper-based and non-copper-based electrocatalysts reported by numerous researchers, with the copper-based electrocatalysts consisting of three main components: copper-nitrogen-doped carbon materials, oxide-derived copper, and copper alloys. Finally, a visual comparison of the selectivity of different electrocatalysts for the production of ethanol from eCO_2_RR is presented through the data in Table 1.

**Table 1:** Summary of eCO_2_RR-to-ethanol performance over copper-based and non-copper-based electrocatalysts

Electrocatalysts	Electrolyte	Potential (V vs. RHE)	Product	FE (%)	Partial current density (mA cm^−2^)	Electrode	References
*Copper-Based Carbon Supported Electrocatalysts*							
Cu_0.5_NC	0.1 M CsHCO_3_	− 1.2	C_2_H_5_OH	55	− 16.2	“Flow” conditions	[75]
CuOCu–N_4_	1 M KOH	− 0.30	C_2_H_5_OH	56.3	− 10.76	Flow cell	[110]
Cu/N_0.14_C	0.1 M KHCO_3_	− 1.1	C_2+_ (C_2_H_5_OH)	73 (51)	− 14.4	H- type cell	[111]
Cu–N-G	0.1 M KHCO_3_	− 0.8	C_2_H_5_OH	33.1	–	H-type cell	[114]
Cu-SACs-N-CQDs	0.1 M KHCO_3_	− 0.2	C_2_H_5_OH	70	1.0	H-type cell	[74]
Cu/CNS	0.1 M KHCO_3_	− 1.2	C_2_H_5_OH	63	− 2.7	H-type cell	[185]
Cu/C-0.4	0.1 M KHCO_3_	− 0.7	C_2_H_5_OH	~ 91	–	H-type cell	[28]
*Oxide derived copper electrocatalysts*							
dCu_2_O/Ag_2.3%_	1 M KOH	− 0.87	C_2_H_5_OH	40.8	326.4	Flow cell	[121]
se-Cu_2_O/Ag	0.1 M KHCO_3_	− 1.1	C_2+_ (C_2_H_5_OH)	60.8 (46.3)	417	H-type cell	[123]
Al–Cu/Cu_2_O	1 M KOH	− 1.0	C_2+_ (C_2_H_5_OH)	84.5 (55.2)	354.2	Flow cell	[60]
Cu_2_O–TiO_2_	0.5 M KHCO_3_	− 0.7	C_2_H_5_OH	27.13	–	H-type cell	[129]
CuO_x_@C	1 M KOH	− 1.0	C_2+_ (C_2_H_5_OH)	82 (46)	166	Flow cell	[133]
Cu/Cu_2_O–CV	0.1 M KHCO_3_	− 1.06	C_2+_ (C_2_H_5_OH)	73.44 (56.56)	–	H-type cell	[27]
*Bimetallic electrocatalysts*							
CuSn–HAB	1 M KOH	− 0.57	C_2_H_5_OH	56(2)	68	Flow cell	[66]
Cu_2_Mg(111)	1 M KOH	− 0.84	C_2_H_5_OH	76.2 ± 4.8	600	Flow cell	[145]
CuAg-0.75%	1 M KOH	− 0.71	C_2_H_5_OH	21.0	214.4	Flow cell	[159]
Cu_1.22_V_0.19_Se	0.1 M KHCO_3_	− 0.8	C_2_H_5_OH	68.3	− 207.9	H-type cell	[162]
K_11.2%_–Cu_2_Se	0.1 M KHCO_3_	− 0.8	C_2_H_5_OH	70.3	97.6	H-type cell	[61]
Cu/AgIOs	0.2 M KHCO_3_	− 1.05	C_2_H_5_OH	29.5	− 16.8	H-type cell	[186]
Cu(Ag-20)_20_	0.1 M KHCO_3_	− 1.1	C_2+_ (C_2_H_5_OH)	31.4 (16.5)	− 4.14	H-type cell	[157]
Cu/Au	1 M KOH	− 0.75	C_2_H_5_OH	60	− 300	Flow cell	[59]
Co_0.050_-Sub-CuS	1 M KOH	− 0.8	C_2_H_5_OH	80	602.0	Flow cell	[187]
*Non-copper-based electrocatalysts*							
SnS_2_/Sn_1_-O_3_G	0.5 M KHCO_3_	− 0.9	C_2_H_5_OH	82.5	17.8	H-type cell	[67]
Ag_0.015_ In_0.985_Se_0.734_	0.1 M KHCO_3_	− 0.6	C_2_H_5_OH	75.2	13.4	H-type cell	[171]
Co-TAPA-OPE COF	0.2 M KHCO_3_	− 0.67	C_2_H_5_OH	66.8	2.87	H-type cell	[177]
PAF-PA5-Ag-0.8	0.1 M KHCO_3_	− 1.0	C_2_H_5_OH	55	11	H-type cell	[181]
Ag/Ag_2_S NWs	0.5 M KHCO_3_	− 0.95	C_2_H_5_OH	75	–	H-type cell	[182]
Ni@NCNT	0.5 M KHCO_3_	− 0.5	C_2_H_5_OH	38.5	–	H-type cell	[184]

**Fig. 16:**
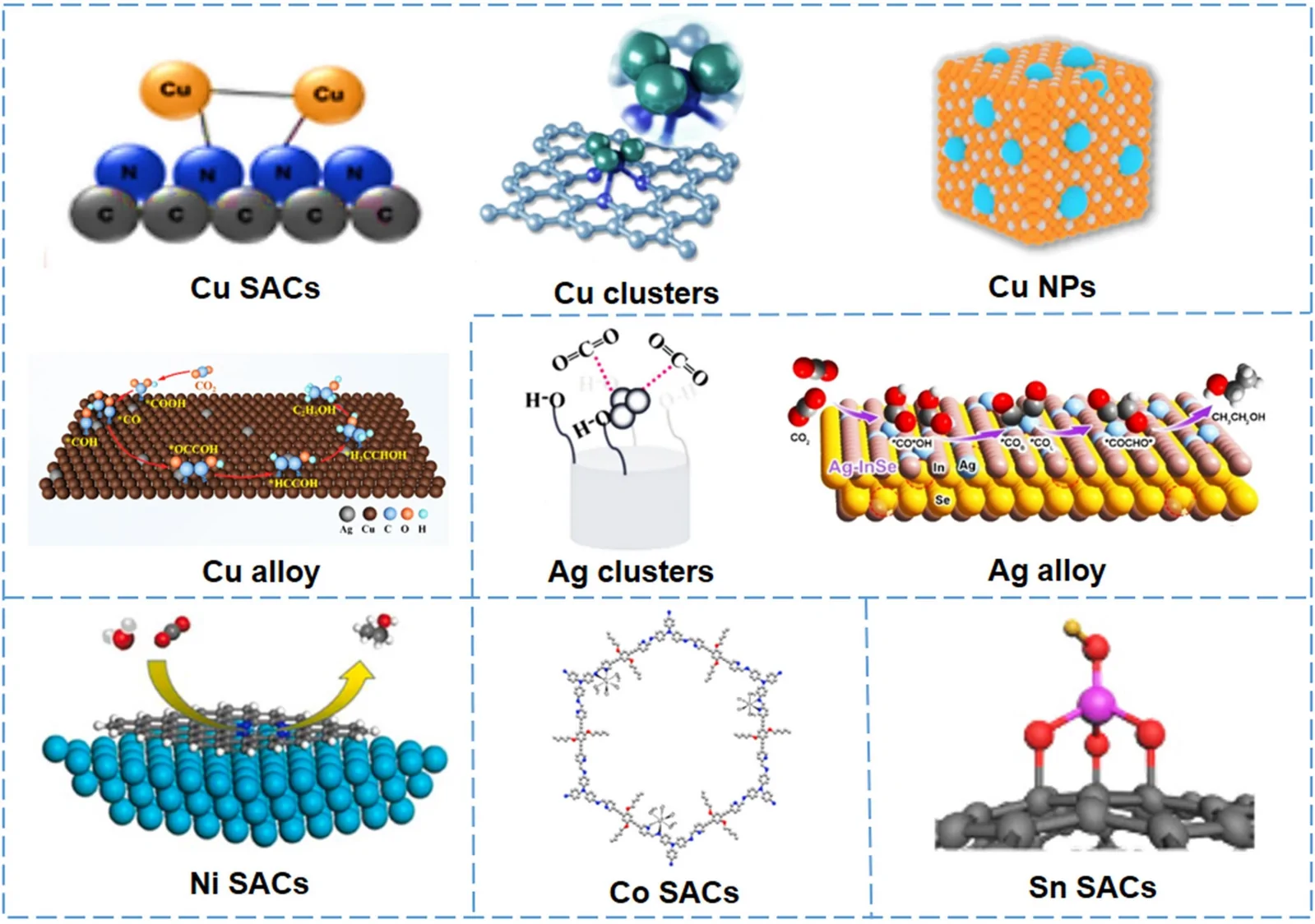
Elements have been identified that can act as dimerization sites to facilitate ethanol production

Through the discussion in this paper, we intend to give readers with comprehensive experimental conditions and findings for the preparation of ethanol by eCO_2_RR. As shown in Fig. 16, a visual categorization of the different elements that can provide dimerization sites during the formation of ethanol was also generated. So far, the electrocatalysts for ethanol production by eCO_2_RR, despite substantial advancements in activity and selectivity, Cu-based electrocatalysts continue to face two fundamental challenges: (1) the inherent product diversity of eCO_2_RR leading to mixed output streams, and (2) insufficient stability or scalability for direct industrial implementation [183]. Building on these findings, we propose several strategic directions for advancing efficient eCO_2_RR electrocatalysts and systems toward ethanol production.

1. Regulating the crystallographic orientation, particle size, and surface roughness of copper catalysts is a pivotal strategy to enhance ethanol selectivity. First, exposing specific facets like Cu(110) or Cu(751) is crucial as they favor the formation of oxygenated products over ethylene [188–190]. Second, particle size must be carefully controlled: Below 2 nm, selectivity shifts overwhelmingly toward H_2_ and CO, while sizes above 10 nm are generally more favorable for C_2+_ products like ethanol. Third, increased surface roughness promotes local OH^−^ concentration, which can facilitate the C–C coupling step. However, a key challenge for practical application is that these engineered features—crystal facets, particle size, and roughness—are susceptible to dynamic reconstruction under operating conditions, which can alter selectivity or cause deactivation. Therefore, if this strategy is to be practically applied in the process of eCO_2_RR ethanol production, it is also necessary to ensure the stability of crystal surface and morphology.
2. The deliberate introduction of defects represents a crucial strategy for enhancing electrocatalytic activity and ethanol selectivity in CO_2_ reduction reactions [191]. Grain boundaries serve as primary catalytic sites for the electrochemical conversion of CO_2_ to multicarbon products. The unsaturated coordination environments inherent to these boundaries facilitate stronger CO_2_ adsorption and lower the energy barrier for CO* dimerization, thereby promoting the formation of multicarbon products such as ethanol. Furthermore, heteroatom incorporation offers additional avenues for performance optimization. Metal doping, for instance, induces strain and ligand effects within copper electrocatalysts, creating adjacent copper sites with distinct electronic configurations. These modified sites enable differentiated C_1_-C_1_ and C_1_-C_2_ coupling pathways, potentially yielding ethanol and longer-chain carbon products. Collectively, these defect engineering approaches—through grain boundary optimization and heteroatom doping—constitute a promising framework for developing advanced electrocatalysts with improved activity, selectivity, and operational stability for sustainable ethanol production.
3. Electron-donating functional groups anchored to copper surfaces effectively modulate the adsorption configuration of *CO intermediates, enabling enhanced selectivity toward oxygenated hydrocarbons during CO_2_ electroreduction [192]. Contemporary studies demonstrate that nitrogen-containing groups in particular significantly boost C_2+_ product selectivity on Cu-based electrocatalysts. These functionalities donate electron density to copper active sites, thereby strengthening *CO adsorption and activating the kinetically challenging C–C dimerization process. Furthermore, molecular modification strategies that elevate local *CO coverage on copper surfaces have emerged as effective approaches for improving ethanol selectivity. Such modifications exert precise control over intermediate concentration and spatial distribution, creating optimized microenvironments for selective C─C coupling. This molecular-level engineering provides substantial flexibility for tailoring copper electrocatalyst selectivity in eCO_2_RR systems.
4. Optimizing the catalytic microenvironment and electrolyzer architecture represents a crucial strategy for maximizing eCO_2_RR performance [193]. While current research predominantly focuses on electrocatalyst materials, systematic investigation of external factors, including electrolyzer design and electrolyte composition, is urgently needed. The electrolyte’s ionic species induce differential polarization effects on CO intermediates, modulating their adsorption strength on electrocatalyst surfaces. Simultaneously, solution pH governs proton availability and CO coverage, thereby dictating C–C coupling kinetics. CO_2_ partial pressure further influences dissolution kinetics and mass transport efficiency. Consequently, environmental parameter optimization is essential for enhancing eCO_2_RR selectivity. Beyond conventional H-cells (usually limited to ~ 20 mA cm^−2^), flow cells with gas diffusion electrodes (GDEs) have emerged as industrially relevant platforms. These systems enable gas–liquid phase separation while enhancing local CO_2_ concentration through hydrophobic microstructures. By operating at elevated pH, flow cells simultaneously suppress hydrogen evolution and promote C_2+_ product formation, such as ethanol. Despite achieving industrially relevant current densities at low overpotentials in flow cells, challenges persist regarding: GDE flooding exacerbating HER and impeding CO_2_ transport; product separation complexity in multiphase systems. Membrane electrode assemblies (MEAs) address these limitations by replacing liquid electrolytes with solid electrolytes. This configuration enables precise tuning of catalyst–membrane interfacial properties, significantly improving selectivity while preventing CO_2_ crossover. Moreover, MEAs facilitate continuous liquid product extraction. As technology advances, MEA-based systems operating at high current density will dominate future eCO_2_RR research for scalable C_2+_ production.

This review aims to provide a strategic framework and inspire researchers to develop next-generation electrocatalysts for selective ethanol production via eCO_2_RR. We anticipate that ongoing advancements in electrocatalyst design, reactor engineering, and process optimization will accelerate the industrial implementation of CO_2_-to-ethanol conversion technologies.
